# Deep treasury management for banks

**DOI:** 10.3389/frai.2023.1120297

**Published:** 2023-03-22

**Authors:** Holger Englisch, Thomas Krabichler, Konrad J. Müller, Marc Schwarz

**Affiliations:** ^1^Department of Treasury, Thurgauer Kantonalbank, Weinfelden, Switzerland; ^2^Centre for Banking and Finance, Eastern Switzerland University of Applied Sciences, St. Gallen, Switzerland; ^3^Master Programme UZH ETH in Quantitative Finance, Zürich, Switzerland; ^4^Entris Banking, Berne, Switzerland

**Keywords:** Asset Liability Management (ALM), deep hedging, deep stochastic control, dynamic strategies, machine learning in finance, reinforcement learning, term structure modeling

## Abstract

Retail banks use *Asset Liability Management* (ALM) to hedge interest rate risk associated with differences in maturity and predictability of their loan and deposit portfolios. The opposing goals of profiting from maturity transformation and hedging interest rate risk while adhering to numerous regulatory constraints make ALM a challenging problem. We formulate ALM as a high-dimensional stochastic control problem in which monthly investment and financing decisions drive the evolution of the bank's balance sheet. To find strategies that maximize long-term utility in the presence of constraints and stochastic interest rates, we train neural networks that parametrize the decision process. Our experiments provide practical insights and demonstrate that the approach of Deep ALM deduces dynamic strategies that outperform static benchmarks.

## 1. Introduction

### 1.1. Background

Recently, deep learning-based techniques have successfully been applied to stochastic control problems in finance. As opposed to classical approaches that rely on the analytical tractability of the problem, recent approaches such as *deep stochastic control* feature a high flexibility. Intricate impediments such as constraints, frictions, and arbitrarily complex stochastic dynamics can be accounted for without further ado. The field of Asset Liability Management (ALM) can particularly profit from the flexibility of this new modeling paradigm. In the context of retail banking, ALM has the task of managing the bank's interest rate risk, which arises from the maturity mismatch of loans and deposits. To this end, banks invest their customers' funds, raise money to finance lending, and enter into interest rate derivatives such as swaps. At the same time, banks have to adhere to regulatory constraints and follow several concurrent objectives. ALM is consequently a challenging problem to both model and solve. This article approaches these two tasks: we develop a modeling framework for ALM and use deep learning techniques to find optimal investment and financing decisions.

**Figure 1:**
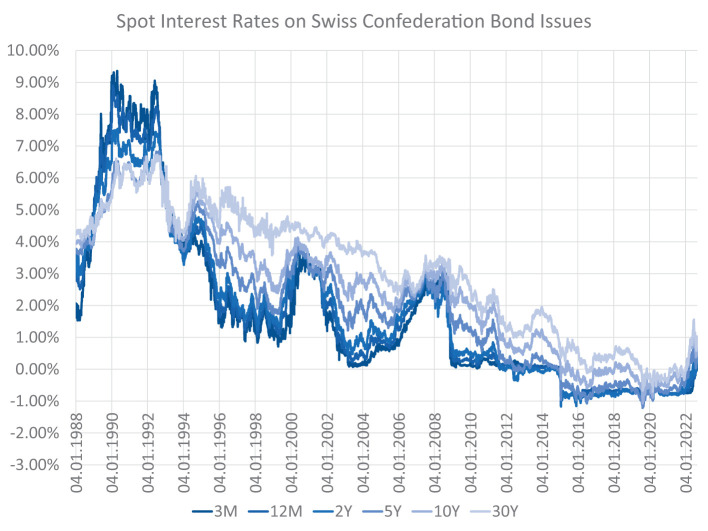
Historical CHF term structures—The optimal balance sheet structure and the interest rate exposure of a bank highly depend on the current and future states of the yield curve. Historically, the term structure featured extremely high and inverted yields in the early 90s. Since the mid 90s, there has been a long-term trend of falling yields with presumed trend reversal during the year 2022.

Retail banks face interest rate risk because cash flows that originate from their loans on the asset side and deposits on the liability side differ in terms of their maturity structure and predictability. If the term structure of interest rates changes, the economic value of the bank's assets might change to a different extent than that of its liabilities, leading to a change in the net position: the bank's equity. Banks do not want to be susceptible to the volatility of interest rates and use ALM to reduce interest rate risk. This involves reducing the discrepancy in the cash flow characteristics of assets and liabilities, and modeling how interest rates might change in future. Yield curve modeling becomes particularly important when applying deep learning-based techniques to the problem. These techniques are 'data-hungry' in the sense that their optimization requires a large bundle of scenarios that specify how interest rates might evolve in future. For this purpose, this article discusses different models for yield curve simulation including a method that generates a variety of yield curve shapes and paths within the HJM framework; see Heath et al. (1992). Figure 1 depicts the historical development of the CHF yield curve^1^ over the last couple of decades.

The yield curve scenarios are used to optimize an ALM strategy that is parametrized with neural networks (*Deep ALM*). This deep learning-based optimization approach, as presented by Han and Weinan (2016) in a general stochastic control setting, is motivated by the success of *deep hedging* (Buehler et al., 2019), which uses neural network-based strategies to hedge financial derivatives. Deep ALM focuses on the problem of hedging interest rate risk of the asset and liability portfolios of banks. In the case of hedging a runoff portfolio, Krabichler and Teichmann (2020) demonstrate that their deep learning-based strategy outperforms a static replication approach as commonly used in practice. This article expands on their approach of hedging a single portfolio and applies deep stochastic control in a more comprehensive model of the ALM problem. This comprises bond portfolios on either side of the balance sheet, decisions on investments and financing, and more realistic constraints. The Deep ALM framework has been developed in collaboration with a Swiss retail bank, hereafter simply referred to as *the* bank. Because the numerical experiments use data provided by the bank, results are sometimes presented in aggregation or on a relative scale.

### 1.2. Asset liability management

The core business of retail banks consists of borrowing and lending funds from and to customers at a variety of maturities. This means that a majority of the bank's assets and liabilities, the so-called *banking book*, consists of long and short positions in future cash flows. The economic value^2^ of this portfolio is given by discounting the cash flows based on the current term structure of interest rates. The value of a bank is thereby largely driven by the external factor of the prevailing *yield curve*, which can be quite volatile. It is not in the interest of banks and their investors that equity as the net position of assets and liabilities is susceptible to a high market volatility. Instead, banks aim to hedge this interest rate risk. This is the core responsibility of ALM. At the same time, banks often keep some exposure to interest rate risk, which allows them to profit from upward slopes in the yield curve. Managing this exposure while adhering to constraints and expectations from different stakeholders makes ALM a challenging problem.

Interest rate risk occurs because cash flows from assets and liabilities differ in several characteristics. First, cash flows occur at different times, and contracts are entered into for different maturities. For instance, mortgages are usually granted for long maturities while deposits are a source of short-term financing. This maturity mismatch leads to a *duration gap* between assets and liabilities. A second fundamental difference lies in the predictability of future cash flows. For most assets in the banking book, banks know what future interest payments they supposedly receive. For instance, interest payments of fixed-rate mortgages are determined when the mortgage is granted to the customer. On the other side of the balance sheet, future interest rates on deposits are unknown. They relate to market interest rates (such as interbank rates) through competition between banks. If interbank rates increase, some banks will offer higher interest rates to their customers, forcing other banks to follow until an equilibrium is reached. In times of positive interest rates, this equilibrium rate is typically lower than that from the interbank market. During the recent negative interest rates regime, customer rates were often floored at 0%, implying that customers essentially held a real option on interest rate payments. Furthermore, most deposits are not placed for a fixed maturity and can be withdrawn by customers at any time. Regarding *non-maturing deposits*, future interest rates are not only unknown but also the timing of when the notional becomes due. This imbalance of deterministic (or at least 'foreseeable') cash flows from assets and stochastic cash flows from deposits is one of the key challenges of ALM.

A common ALM approach for hedging interest rate risk is found on the notion of *replicating portfolios*. Liabilities in the banking book with undetermined cash flows are invested in a bond portfolio that *replicates* the interest rate sensitivity of the liability portfolio, such that the net interest rate risk is minimal. Similarly, assets with undetermined interest rate payments can be financed with matching replicating portfolios. The difficulty of this approach lies in selecting a suitable mix of maturities in the replicating portfolios. For instance, to replicate the deposit position, the bank should choose maturities such that the interest earned on the replicating portfolio moves parallel with the interest paid to customers. The risk of rising interest rates can be mitigated by investing in short maturities; higher interest payments on deposits can be financed from the replicating portfolio that is renewed continually. Nonetheless, investing in longer maturities usually offers higher yields (at the cost of a more pronounced interest rate risk).

Banks typically keep some interest rate exposure to exploit spreads that banks charge customers when lending and borrowing money. Most often, banks keep a higher *duration*^3^ on their assets than on their liabilities; long-term investments through rolled over short-term funding. If the yield curve features a positive slope, it allows banks to lend funds at the far end of the curve while borrowing funds at short maturities with smaller rates. If yields stay relatively constant over time, this *carry* trade generates a profit for the bank. But this *maturity transformation* is subject to the risk of rising interest rates. If the yield curve shifts upwards, refinancing at the short end becomes more expensive while the interest earned on previously issued loans remains unaffected. This leads to an obliteration of projected revenue.

### 1.3. Deep learning for stochastic control problems

The following introduces a general stochastic control problem in discrete and finite time with time instances *t* ∈ {0, 1, 2, …, *T*} on the filtered probability space (Ω,F,F,ℙ). The observable information that characterizes the control problem at time *t* is summarized *via* the $F_{t}$-measurable and *d*-dimensional *state* variable *x*_*t*_. The state *x*_*t*_ evolves to state *x*_*t*+1_ according to a transition function *b*_*t*_. If the control problem is Markovian, as in the setting of Han and Weinan (2016), *b*_*t*_ maps the current state $x_{t}\in {\mathbb{R}}^{d}$, the control $a_{t}\in {\mathbb{R}}^{m}$, and a random shock ε_*t*+1_ to the next state *x*_*t*+1_. We assume a slightly more general setting, where the transition might also depend on the history *h*_*t*_ of the previously attained states.^4^ At each time step, the function $u_{t}:{\mathbb{R}}^{d}\times {\mathbb{R}}^{m}\to \mathbb{R}$ assigns utility or a reward with the current state-action pair. The optimization aims to optimize the cumulative utility in expectation while respecting potential inequality constraints $g_{i}:{\mathbb{R}}^{d}\times {\mathbb{R}}^{m}\to \mathbb{R}$ and equality constraints $k_{i}:{\mathbb{R}}^{d}\times {\mathbb{R}}^{m}\to \mathbb{R}$. In summary, this gives the stochastic control problem


(1a) $$\begin{array}{l} \underset{{\left\{a_{t}\right\}}_{t=0,1,\ldots ,T-1}}{\max}\;\text{E }\left[\overset{T-1}{\underset{t=0}{\sum }}u_{t}\left(x_{t},a_{t}\right)+u_{T}\left(x_{T}\right)\right] \end{array}$$

(1b) $$\begin{array}{l} \text{subject to }x_{t+1}=b_{t}\left(h_{t},x_{t},a_{t},\varepsilon_{t+1}\right), \end{array}$$

(1c) $$\begin{array}{l} \;h_{t}=\left\{x_{0},x_{1},\ldots ,x_{t-1}\right\}, \end{array}$$

(1d) $$\begin{array}{l} \;g_{i}\left(x_{t},a_{t}\right)=0,\;\forall \;i=1,2,\ldots ,I, \end{array}$$

(1e) $$\begin{array}{l} \;k_{j}\left(x_{t},a_{t}\right)\leq 0,\;\forall \;j=1,2,\ldots ,J. \end{array}$$

*Deep learning* can be used to approximately solve stochastic control problems. By parametrizing controls with neural networks, these controls can be optimized using gradient descent. This method, hereafter referred to as *deep stochastic control* (DSC)^5^, is the basis of deep hedging (Buehler et al., 2019), deep replication (Krabichler and Teichmann, 2020), and the Deep ALM approach developed in this article.

Let *L, N*_1_, *N*_2_, …, *N*_*L*_ ∈ ℕ with *L* ≥ 2, σ:ℝ → ℝ and let $W_{l}:{\mathbb{R}}^{N_{l-1}}\to {\mathbb{R}}^{N_{l}}$ be an affine function. A *feedforward neural network* is a function $g:{\mathbb{R}}^{N_{0}}\to {\mathbb{R}}^{N_{L}}$ such that


$$\begin{array}{l} g\left(x\right)=W_{L}\circ g_{L-1}\circ \cdots \circ g_{1},\\ \;g_{l}=\sigma \circ W_{l},\\ \;W_{l}=A^{l}x+b^{l}, \end{array}$$

for the layers *l* = 1, 2, …, *L*−1. The *activation function* σ is applied componentwise. The entries of the matrices {*W*_*l*_}_*l* = 1, 2, …, *L*−1_ and vectors {*b*_*l*_}_*l* = 1, 2, …, *L*−1_ are called the *weights* of the neural network. These weights are referred to as θ and the dependence of the neural network on its weights is highlighted *via* the notation *g*^θ^.

The key idea behind DSC is to parametrize the action *a*_*t*_ at each time instance *t* with a neural network $g^{\theta_{t}}$ that determines the action based on the relevant and available information at time *t*. Assuming this information can be captured by the state *x*_*t*_ and a memory cell $h_{t}\in {\mathbb{R}}^{t}$, the neural network maps the concatenated input to the action space, i.e., $g^{\theta_{t}}:{\mathbb{R}}^{d+t}\to {\mathbb{R}}^{m}$. The objective (Equation 1a) can now be formulated as a maximization over the parameters of all neural networks ${\left\{g^{\theta_{t}}\right\}}_{t=0,1,\ldots ,T-1}$, i.e.,


(2) $$\begin{array}{l} \underset{{\left\{\theta_{t}\right\}}_{t=0,1,\ldots ,T-1}}{\max}\text{E }\left[\overset{T-1}{\underset{t=0}{\sum }}u_{t}\left(x_{t},g^{\theta_{t}}\left(x_{t},h_{t}\right)\right)+u_{T}\left(x_{T}\right)\right]. \end{array}$$

Assuming knowledge and differentiability of the transition dynamics {*b*_*t*_}_*t* = 0, 1, …, *T*−1_, the optimization can be approached using techniques based on gradient descent. First, parameters {θ_*t*_}_*t* = 0, 1, …, *T*−1_ are initialized randomly. Subsequently, given the initial state *x*_0_ and the ability to sample the random shocks {ε_*t*_}_*t* = 1, 2, …, *T*_, one can collect complete roll-outs, i.e., paths of states, actions, and rewards that have occurred over the entire model period. This is achieved by chaining the forward passes through the decision networks ${\left\{g^{\theta_{t}}\right\}}_{t=0,1,\ldots ,T-1}$ as well as the transitions {*b*_*t*_}_*t* = 0, 1, …, *T*−1_ in their temporal order. Utilizing the collected rewards {*u*_*t*_}_*t* = 1, 2, …, *T*_, one can calculate a loss signal for each path that is backpropagated through the entire computational graph.

Simply optimizing for the cumulative reward would generally neglect the constraints of the stochastic control problem, if they are not accounted for otherwise.^6^ In that case, one prevalent approach for dealing with constraints is to consider negative reward signals, whenever those are violated. The cumulative loss or cost until and including time *t* is then given by


(3) $$\begin{array}{l} C_{t}\;:\;=\sum_{\tau =0}^{t}\{-u_{\tau }(x_{\tau },g_{\theta_{\tau }}(x_{\tau },h_{\tau }))+\sum_{i=1}^{I}\lambda_{i}P_{e}(g_{i}(x_{\tau },a_{\tau }))\\ \;+\sum_{j=1}^{J}\sigma_{j}P_{ie}(k_{j}(x_{\tau },a_{\tau }))\} \end{array}$$

for suitable penalty weights λ, σ_*j*_ ≥ 0 and penalty functions *P*_*e*_(·) and *P*_*ie*_(·) that monitor the occurrence and magnitude of breaches. The final loss signal to be minimized is then given by *C*_*T*_.

**Figure 2:**
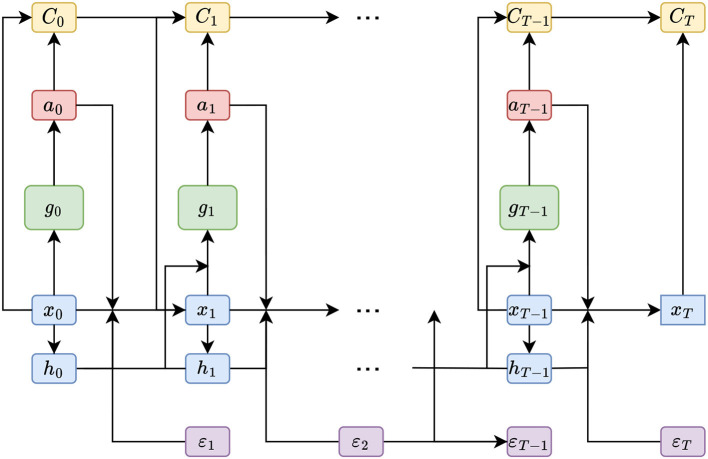
Computational flow (DSC)—This figure illustrates the order of computations made in the DSC algorithm to reach the final state *x*_*T*_ from the initial state *x*_0_. The figure is largely based on Figure 1 from Han and Weinan (2016), but extended by the memory cells *h*_0_, *h*_1_, …, *h*_*T*−1_.

Han and Weinan (2016) illustrate that the concatenated computations to calculate a single roll-out can be regarded as a single deep neural network where the transitions {*b*_*t*_}_*t* = 0, 1, …, *T*−1_ are differentiable layers without trainable parameters (see Figure 2). As outlined later, it might make sense to share weights between the neural networks, i.e., setting $g^{\theta_{t}}\equiv g^{\theta }$. In that case, the computational graph reminds one of the computations in a *recurrent* neural network with the addition of the transition layer. In this context, backpropagating the final error signal by unfolding the recurrent structure (as illustrated in Figure 2) is referred to as *backpropagation through time*; see Werbos (1990). This connection becomes noticeable when applying DSC to ALM, where gradients are found to be vanishing (see, Hochreiter, 1998). This is a common problem in training recurrent neural network architectures and is not surprising considering the computational similarities.

**Figure 3:**
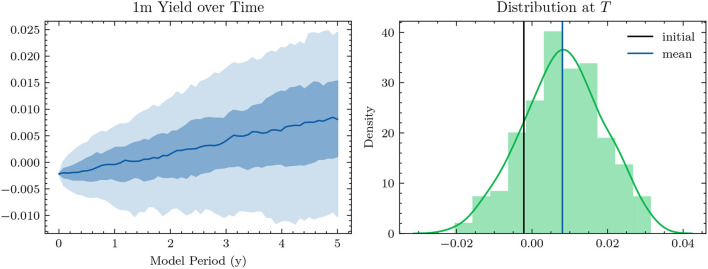
Simulated 1*m*-Yields (HJM-PCA)—In the plot on the left-hand side, the solid line represents the median 1*m*-yield over all scenarios. The darker shaded area is enclosed by lines representing the 25% quantile and the 75% quantile. The lighter shaded area is enclosed by the 5% quantile and the 95% quantile.

Generally speaking, *reinforcement learning* is about training agents to execute action sequences that maximize cumulative rewards in a possibly non-continuous, non-differentiable, partially observable environment (see, Faccio et al., 2022). In this sense, the stochastic control setting presented earlier is a reinforcement learning problem with the particularity that the dynamics of the environment are known and differentiable. The lack of these two properties motivates most of reinforcement learning, where the environment is either assumed to be a black box (*model-free* paradigm) or has to be learnt (*model-based* paradigm). Being able to immediately take the gradient of the reward with respect to the policy's parameters eliminates the need to reparametrize the gradient (Williams, 1992) or resort to purely correlating actions with rewards; see Lillicrap and Santoro (2019) for a discussion.

### 1.4. Machine learning in finance

Particularly for applications arising in finance, it is certainly meaningful to tackle intricate optimization problems with the efficiency and natural simplicity of the DSC algorithm. It can be implemented by utilizing the amenities of automatic differentiation engines in modern deep learning libraries that handle all gradient computations. Instead of solving the full problem for all points in time and space (and being exposed to the so-called *curse of dimensionality*, under which the running time of an algorithm grows exponentially in the number of dimensions), one learns a convincing strategy with respect to an initial state *x*_0_ and a bundle of scenarios. This trades off generality as models have to be retrained once the initial state, the transition logic, or the scenario generator have changed. Whereas, this might be undesirable in some applications, e.g., when deep-hedging many different derivatives on different underlyings issued recurringly (Buehler et al., 2022b), it is less problematic in the case of Deep ALM since, in practice, there is indeed only one entity subject to a single initial state.

Deep hedging (Buehler et al., 2019) has received significant attention from both academics and practitioners because it often outperforms classical methods relying on tractable models. As opposed to the classical modeling and problem-solving paradigm, deep hedging offers a generic approach for finding an approximately optimal hedging strategy: parametrization of the hedging strategy with neural networks and training thereupon to minimize the hedging error on a set of simulated market trajectories. The modeling task is split into a simulation task and an optimization task, that is conditional on the simulated paths. This allows for using all kinds of techniques for generating scenarios; e.g., see Buehler et al. (2020) and Wiese et al. (2020). The flexibility of the deep hedging framework does not only lie in the choice of the market simulator but also in the simple adaptability to arbitrary (possibly path-dependent) payoffs and in the extensibility to account for market frictions such as transaction cost and liquidity squeezes. Even a fundamental change to the problem such as replacing a Markovian market simulator with non-Markovian dynamics can be accounted for by replacing the feed-forward neural networks with recurrent neural networks (see, Horvath et al., 2021).

While most of the work on deep hedging focuses on managing financial derivatives, Krabichler and Teichmann (2020) apply the DSC approach to the problem of funding and hedging a runoff portfolio. Exemplarily, it represents a not yet unwound liability on the balance sheet of a property and casualty insurance company. Practitioners do not want to keep these positions unhedged and look rather for a strategy to maximize risk-adjusted returns of the net portfolio (i.e., equity). Investment decisions become high-dimensional because at each point in time, a whole series of bonds with different maturities is issued along the term structure. Krabichler and Teichmann (2020) demonstrate that applying the DSC approach in due course leads to a dynamic strategy that outperforms a static replication scheme which is commonly used in practice. Since the replication of a bond portfolio is a fundamental task within ALM on either side of the balance sheet for investment and financing decisions, the success of deep replication motivates the application of DSC to a full description of the ALM problem, which we denote as Deep ALM. Incorporating all components of the ALM problem while finding the right balance between all the different goals without adversely affecting the robustness of the learning process entails some engineering work; see Krabichler and Teichmann (2020). In this article, we pursue this engineering work, develop a realistic ALM framework, and apply the DSC approach within it. We expand on the stylized replication problem because we make investment decisions for non-maturing portfolios involving stochastic depreciation, extend the hedging instruments to swaps, and replace the simple liquidity constraint by more realistic counterparts. More precisely, these comprise a minimum reserve, standard liquidity measures, a leverage constraint, risk limits in terms of an interest rate sensitivity, and a minimum target return.

Other ALM problems that have been approached with machine learning and reinforcement learning techniques are different from our setting. There is an extensive literature on the use of deep learning for mere investment portfolio optimization (whereby funding and other intricacies of ALM are not treated); e.g., see Zhang et al. (2020). Fontoura et al. (2019) consider the problem of determining the allocation of funds toward asset classes such that a portfolio of liabilities can be paid off using these assets. Their problem setting is different from ours as they consider a runoff setting (no going concern), only optimize relative investment decisions (not the scale), do not consider financing decisions or constraints, and use a binary objective of whether assets are sufficient to pay all debts or not. Cheridito et al. (2020) use neural networks to approximate the value and thereby the risk of a liability portfolio consisting, for instance, of options or variable annuities. This deviates from our setting since we face asset and liability portfolios that consist mainly of bonds and other deterministic as well as stochastic cash flows.

### 1.5. Structure of the article

Section 2 elaborates on the ALM problem. Subsequently, the Deep ALM approach and its implementation is outlined in Section 3. Section 4 presents the main results and a comprehensive set of in-depth analyses. Section 5 provides a brief summary of the key findings and lists remaining issues before deploying Deep ALM. An overview of all variables and parameters is attached in the Appendix.

## 2. The ALM optimization problem

### 2.1. States and actions

#### 2.1.1. Setting the scene

To apply deep learning techniques to ALM, one needs a comprehensive mathematical description of the *balance sheet roll-forward*. In this section, ALM is formulated as a stochastic control problem. This inevitably involves simplifying assumptions on components of ALM that are potentially much more complex in reality. Many of the simplifications (e.g., deterministic depositing behavior) can be replaced by more complex models in a straightforward manner such that the Deep ALM approach is still feasible. To obtain convincing results after refining the problem setting, additional work such as the incorporation of additional features may well be required.

While many reinforcement learning problems can be formalized as *Markov decision processes* (MDP), we do not frame the ALM problem as an MDP as we do not restrict the transition function to be Markovian. Still, the following sections describe—analogously to the description of an MDP—what variables are modeled (*state*), what decisions can be made (*actions*), how these decisions impact the model state (*transition*), and how given states are evaluated (*rewards*) to optimize the actions. Since we are going to introduce a considerable number of variables, we provide a comprehensive overview of the notation in the Appendix.

This section provides a brief overview of the model variables. They consist of positions on an aggregated and simplified balance sheet of a bank, the so-called *banking book* as specified in Table 1, other variables that impact the bank's income statement, and the yield curve. Each variable is modeled over *H* equidistant time steps *t* ∈ 𝕋: = {0, Δ*t*, …, *T*} with *T* = (*H*−1)Δ*t* on the filtered probability space (Ω,F,F,ℙ), where $\text{F}={\left(F_{t}\right)}_{t\in \text{T}}$. The time discretization is chosen such that the step size Δ*t* = 1/12 corresponds to 1 month. We distinguish between variables representing *nominal* cash flows and variables representing the *value* of a cash flow (i.e., the discounted cash flow). At any time *t*, a nominal cash flow is tracked up to *N* time steps Δ*t* into the future and modeled as an *N*-dimensional (random) vector, where the *i*^*th*^ entry refers to a cash flow at the global time step *t*+*i*, i.e., a cash flow being due after *i* steps when viewed from time *t*. A cash flow being settled *i* time steps has consequently a maturity of τ = *iΔt* and the set of all maturities is denoted as T:={Δt,…,NΔt}. Some of the nominal cash flows are assumed to be deterministic, while all discounted cash flows are random variables due to their dependence on the yield curve.

**Table 1:** Economic balance sheet—Loans and deposits are demand-driven, whereas investments and borrowings are subject to the control of the bank.

**Assets**	**100*%***	***A*_*t*_**	**Liabilities (without Equity)**	**90*%***	***L*_*t*_**
Cash	20%	*C*_*t*_	Deposits	50%	*V*(*S*_*t*_)
Investments	5%	*V*(*B*_*t*_)	Non-Maturing Deposits	10%	$V\left(S_{t}^{D}\right)$
Loans	75%	*V*(*R*_*t*_)	Term Deposits	40%	$V\left(S_{t}^{F}\right)$
Mortgages	55%	$V\left(R_{t}^{P}\right)$	Borrowings	40%	*V*(*K*_*t*_)
Loans to Enterprises	20%	$V\left(R_{t}^{E}\right)$	**Equity**	10%	*E*_*t*_

The relative weights roughly specify the initial distribution. The loan portfolio includes positions with a maturity of up to 15*y*, but the majority of loans have a much shorter maturity such that the loan portfolio has a duration of fewer than 5*y*. Deposits are modeled with maturities of up to 10*y*, but most of the deposits are associated with a much shorter maturity. The deposit portfolio has a duration of fewer than 3*y*. The exact composition of these portfolios is not reported for data privacy.

#### 2.1.2. Yield curve and discount factors

In our formulation of the ALM optimization problem, the yield curve is the most important component because it is the only source of randomness. The yield curve determines bond prices (or rather coupons), discounted values of nominal cash flows, as well as additional effects on the bank's income such as depreciation and penalties on cash. The yield curve is modeled as the random vector $Y_{t}:\Omega \to {\mathbb{R}}^{N}$, where the *i*^*th*^ entry of *Y*_*t*_ denotes the yield prevailing at time *t* for a maturity of *i*Δ*t*. We sometimes refer to this yield as *Y*(*i*Δ*t*). As the yield curve is the single source of randomness in this model, it is $F_{t}$-measurable by the definition of $F_{t}:=\sigma \left(Y_{0},Y_{1},\ldots ,Y_{t}\right)$. The yield curve *Y*_*t*_ determines the discount factors $D_{t}:\Omega \to {\mathbb{R}}^{N}$ for all maturities τ∈T as


(4) $$\begin{array}{l} D_{t}\left(\tau \right)=e^{-\tau \Delta Y_{t}\left(\tau \right)}. \end{array}$$

The discount factors are used to value nominal cash flows. The value of a nominal cash flow $X_{t}\in {\mathbb{R}}^{N}$ at time *t* is denoted as *V*(*X*_*t*_). It is given by the inner product with the discount factors at time *t*, i.e., *V*(*X*_*t*_) = 〈*D*_*t*_, *X*_*t*_〉.

#### 2.1.3. Balance sheet items

##### 2.1.3.1. Cash and cash equivalents

This position represents all highly liquid assets of a bank. It changes at each model step as loans are issued and paid out, deposits are posted and withdrawn, and costs are settled. It decreases with additional bond investments and increases when raised by issuing bonds. It is highly dependent on the exact decisions made and thus modeled as a random variable *C*_*t*_:Ω → ℝ. The cash position essentially represents cash flows of maturity zero such that *V*(*C*_*t*_) = *C*_*t*_.

##### 2.1.3.2. Loans

The bank issues two types of loans: mortgages and loans to enterprises. The number of new loans that the bank grants each period is assumed to be driven by demand and not influenced by any decision made by the bank. Loan defaults occur whenever the yield curve shifts significantly over a single year. The cash flows of mortgages and loans to enterprises outstanding at time *t* are modeled as random vectors $R_{t}^{P}:\Omega \to {\mathbb{R}}^{N}$ and $R_{t}^{E}:\Omega \to {\mathbb{R}}^{N}$, respectively. Aggregated loans are referred to as $R_{t}:\;=R_{t}^{P}+R_{t}^{E}.$

##### 2.1.3.3. Investments

We assume that the bank can only invest in bonds. At the beginning of the model period, the bank has a legacy portfolio of bonds. At each model step, the bank has the opportunity to invest in several newly issued bonds with different maturities up to a maturity of *N* steps. We assume that bonds cannot be sold (including *no short-selling*) and are always *held-to-maturity*. The aggregated cash flows of all outstanding bonds the bank has invested in up to and including time *t* are denoted by the random vector $B_{t}:\Omega \to {\mathbb{R}}^{N}$. We further distinguish this bond portfolio *B*_*t*_, that includes payoffs from bonds bought in period *t*, from the bond portfolio $B_{t}^{\text{pre}}:\Omega \to {\mathbb{R}}^{N}$ which does not include payoffs from the period *t* investments.

##### 2.1.3.4. Deposits

Customers can make two types of deposits: non-maturing and term deposits. Deposits are assumed to be driven by deterministic demand and not influenced by decisions made by the bank.^7^ While cash flows originating from term deposits naturally have a maturity associated with them, cash flows from non-maturing deposits technically do not have a maturity. Customers can simply withdraw their money whenever they want.^8^ At the same time, it is unlikely that all non-maturing deposits are withdrawn in any single period. Hence, we assume a maturity structure for the non-maturing deposits. “Outstanding” cash flows from non-maturing deposits at time *t* can then be modeled as a deterministic vector $S_{t}^{D}\in {\mathbb{R}}^{N}$. Similarly, cash flows from term deposits due at time *t* are given by $S_{t}^{F}\in {\mathbb{R}}^{N}$, and aggregated deposits $S_{t}\in {\mathbb{R}}^{N}$ are defined as $S_{t}:\;=S_{t}^{D}+S_{t}^{F}$.

##### 2.1.3.5. Financing

In addition to the funding from deposits, we assume that the bank can only raise additional capital by issuing bonds, which are modeled analogously to those on the asset side. Correspondingly, the bank has at each model step the opportunity to issue several new bonds and to add them to its existing financing portfolio. The financing portfolio is modeled analogously to the investment portfolio. Financing positions are always held-to-maturity and cannot be unwound prematurely. The random vector $K_{t}^{\text{pre}}:\Omega \to {\mathbb{R}}^{N}$ denotes the aggregated cash flows originating from all outstanding bonds, that the bank has issued before time *t*, and $K_{t}:\Omega \to {\mathbb{R}}^{N}$ denotes the financing portfolio including the bonds issued at time *t*.

##### 2.1.3.6. Aggregation

Cash, investments, and loans constitute the bank's *assets*. Its value *A*_*t*_:Ω → ℝ is thus given by *A*_*t*_: = *C*_*t*_+*V*(*R*_*t*_)+*V*(*B*_*t*_). With an abuse of notation, *liabilities* consist of deposits and financing. The value of liabilities is referred to as *L*_*t*_:Ω → ℝ and given by *L*_*t*_: = *V*(*S*_*t*_)+*V*(*K*_*t*_). Consequently, the bank's *equity* *E*_*t*_:Ω → ℝ is the residue *E*_*t*_: = *A*_*t*_−*L*_*t*_. The final value of equity *E*_*T*_ is the quantity that the optimization aims to maximize. Note again that we only keep track of the economic balance sheet and ensure that the balance sheet is indeed balanced under this valuation regime. We leave out other accounting aspects such as *accruals* and *amortized cost*, which are typically considered in ALM depending on the accounting standard and legislation.

#### 2.1.4. Actions

As previously mentioned, the bank faces investment and financing decisions each period. It can invest in *b*^*B*^ bonds and borrow from *b*^*K*^ bonds that all trade at par and have different maturities.^9^ Once bought or issued, bonds must be held until maturity. Short-selling is not allowed, which includes that the bank cannot invest in its own issued bonds. Both investment and financing can be done fractionally. We denote the actions made at time *t* by the vector $a_{t}\in {\mathbb{R}}_{\geq 0}^{b^{B}+b^{K}}$. Its first *b*^*B*^ entries represent the number of bonds bought at each of the available investment maturities, also referred to as $a_{t}^{B}\in {\mathbb{R}}_{\geq 0}^{b^{B}}$. Its last *b*^*K*^ entries represent the number of bonds issued at each of the available financing maturities, also referred to as $a_{t}^{K}\in {\mathbb{R}}_{\geq 0}^{b^{K}}$.

### 2.2. Transition of decision-independent variables

The model state at a given time *t* ∈ 𝕋 is captured by the variables introduced earlier. The next two sections describe how the state transitions from time *t* to the next discretized instance *t*+Δ*t*. In the language of DSC, we specify how the transition function *b*_*t*_ acts on the state *x*_*t*_. Because the state in our setting is quite high dimensional and the transition function is a concatenation of many calculations, we omit this notation in the following. Instead, it is more comprehensible to directly describe the evolution of the model variables that make up the model state. We structure the description of the transitions based on whether the transition of a model variable depends on the decisions or not. For decision-independent variables, transitions can later be calculated outside of the optimization. We start by introducing some notation following Krabichler and Teichmann (2020). Let


(5) $$\begin{array}{l} U:=\left[\begin{array}{cc} 0 & {\text{I}}_{N-1}\\ 0 & 0 \end{array}\right], \end{array}$$

where **0** ∈ ℝ^*N*−1^ is the zero vector and ${\text{I}}_{N-1}\in {\mathbb{R}}^{\left(N-1\right)\times \left(N-1\right)}$ the identity matrix. When applied to an *N*-dimensional vector *X*, *U* shifts all entries up by one, eliminates the first entry, and appends a zero as the new last entry. Moreover, let π^(*k*)^:ℝ^*N*^ → ℝ denote the projection onto the *k*^*th*^ component of an *N*-dimensional vector.

#### 2.2.1. Yield curve, discount factors, and bonds

The transition of the yield curve can generally be given by any term structure model, such as those presented in Section 2.7. Discount factors are then recalculated *via* (Equation 4). In each period, *b*^*B*^ new investment bonds and *b*^*K*^ new financing bonds are issued. Following the setup in Krabichler and Teichmann (2020), bonds pay a semi-annual coupon that is chosen such that bonds trade at par at issuance. The corresponding coupon payments are calculated as follows. For a given investment bond *i* ∈ {1, 2, …, *b*^*B*^} issued at time *t*, we denote its payout structure as ${\tilde{Z_{t}}}^{B,i}\in {\mathbb{R}}^{N}$ with semi-annual coupon $\alpha_{t}^{B,i}\in \mathbb{R}$. Denoting with ${\tilde{Z_{t}}}^{B,i}\left(k\Delta t\right)$ the *k*^*th*^ entry of ${\tilde{Z_{t}}}^{B,i}$, the payout structure is defined for all τ∈T as


(6) $$\begin{array}{l} {\tilde{Z}}_{t}{\;}^{B,i}(\tau )\;:\;=\{\begin{array}{ll} \alpha_{t}^{B,i} & \text{if the bond has coupon payment date at }\tau ,\\ 1+\alpha_{t}^{B,i} & \text{if the bond redeems at }\tau ,\\ 0 & \text{else}. \end{array}\; \end{array}$$

As indicated, $\alpha_{t}^{B,i}$ is chosen such that the bond trades at par, i.e., it is the solution to the linear equation


(7) $$\begin{array}{l} \langle D_{t},{\tilde{Z_{t}}}^{B,i}\rangle \overset{!}{=}1. \end{array}$$

Note that the bank actually receives less than this fair coupon $\alpha_{t}^{B,i}$ on this investment as it faces an annualized spread of κ_*B*_ = −15 bps. ^10^ The cash flow adjusted by spreads is in the following referred to as $Z_{t}^{B,i}$. Financing bonds are treated analogously: the spread-adjusted (κ_*K*_ = 15 bps) cash flow of the *i*^*th*^ financing bond issued at time *t*, where *i* ∈ {1, 2, …, *b*^*K*^}, is referred to as $Z_{t}^{K,i}$.

#### 2.2.2. Loans

The initial loan portfolios for both mortgages and loans to enterprises are provided by the bank and assumed to evolve according to a simple growth scheme. In each period, loans mature leading to repayments of the loaned amount which increases cash. At the same time, new loans ${\tilde{R}}_{t}\in {\mathbb{R}}^{N}$ are granted such that $R_{t+\Delta t}=UR_{t}+{\tilde{R}}_{t}$. Granting new loans leads to a reduction in cash. The total amount of new loans granted in period *t* is assumed to be


(8) $$\begin{array}{l} ∥{\tilde{R}}_{t}{∥}_{1}=\pi^{\left(1\right)}(R_{t})+\frac{\rho_{L}}{12}∥R_{t}{∥}_{1}. \end{array}$$

The loan position grows by slightly more than ρ_*L*_ = 3% per year. The amount of new loans $∥{\tilde{R}}_{t}∥1$ is split over several maturities. For the loans to enterprises, new loans are assumed to be granted equally for maturities of 1*m*–3*m*. New mortgages are attributed to 11 different maturities of 2*y*–12*y* based on a distribution provided by the bank that mimics realistic customer behavior. This distribution is assumed to be deterministic and the same for each model period, which corresponds to the assumption that there is neither stochastic nor interest rate sensitive borrowing behavior of the bank's customers. Mortgages are assumed to be default-free, whereas loans to individuals have some default risk in times of quickly increasing yield curves: at the end of the year, the bank has to depreciate loans to enterprises by a factor of (*k*−2%), if the 6*m* interest rate has increased by *k* > 2% over the past year. The depreciation amount is split proportionally over the current portfolio of loans to enterprises. The dependence of depreciation on the yield curve makes the loan portfolio stochastic.

All loans are assumed to bear fixed interest payments. The monthly interest payment on a loan issued at time *t* with time to maturity τ is calculated based on *Y*_*t*_(τ), i.e., the yield prevailing at time *t* for time to maturity τ. In addition, the bank is assumed to charge its customers an annual spread of κ_*L*_ > 0 and never offers its customers negative interest on loans. The latter assumption is reasonable as most Swiss banks did not offer loans with negative coupons in recent years. Finally, the monthly interest rate payment *r* for a loan granted at time *t* with maturity τ is calculated as


(9) $$\begin{array}{l} r={\left(e^{Y_{t}\left(\tau \right)+\kappa_{L}}-1\right)}^{+}. \end{array}$$

The sum of all interest payments that the bank receives at time *t* on its loans is denoted by *r*_*t*_. For simplicity, interest payments from loans in the legacy portfolio are calculated in the same way using the initial yield curve *Y*_0_, as opposed to calculating them from the yield curve history. Once a loan has been depreciated, it does not pay interest any longer.

#### 2.2.3. Deposits

As mentioned earlier, we assume a maturity structure for non-maturing deposits such that non-maturing and term deposits are treated equivalently from the computational viewpoint. The distribution of deposits over different maturities is simulated *via* a rolling scheme. Each deposit is associated with a maturity of $\tau \in \left\{\frac{1}{12},\frac{1}{6},1,10\right\}$ years. Once a deposit with face amount *A* and reference maturity τ matures, the amount gets reinvested in equal parts into monthly tranches up to the maturity τ. Thus, $\frac{A}{\tau }\Delta t$ gets assigned to each maturity Δ*t*, 2Δ*t*, …, τ in the total deposit portfolio. The initial assignment of deposits to the reference maturities is provided by the bank. In addition, new non-maturing deposits ${\tilde{S}}_{t}^{D}\in {\mathbb{R}}^{N}$ with face amount


(10) $$\begin{array}{l} {\left\|{\tilde{S}}_{t}^{D}\right\|}_{1}=\frac{\rho_{S^{D}}}{12}{\left\|S_{t}^{D}\right\|}_{1}, \end{array}$$

where $\rho_{S^{D}}=4\%$, are placed with the bank. They are assigned to the reference maturities *via* the same distribution used for the initial deposits portfolio. Term deposits increase analogously by ${\tilde{S}}_{t}^{F}\in {\mathbb{R}}^{N}$ with growth rate $\rho_{S^{D}}=1\%$. New total deposits are then given by ${\tilde{S}}_{t}:\;={\tilde{S}}_{t}^{D}+{\tilde{S}}_{t}^{F}$. Interest paid on deposits varies with the level of a reference rate. The latter is defined as the 3*m* moving average of the 6*m*-yield *Y*_*t*_(0.5). This approximates the 6*m*-CHF-OIS, a relevant reference *swap rate* in practice. In addition, the bank imposes caps (and floors) on the paid interest rates depending on the type of the deposit. The time *t* interest rates for non-maturing deposits $u_{t}^{D}$ and term deposits $u_{t}^{F}$ are given by


(11a) $$\begin{array}{l} u_{t}^{D}=\min\{60\%\times {\bar{Y}}_{t}(0.5),3\%\} \end{array}$$

(11b) $$\begin{array}{l} u_{t}^{F}=\min\;\{\max\{85\%\times {\bar{Y}}_{t}(0.5),Y_{t}(0.5)-0.25\%\},5\%\} \end{array}$$

(11c) $$\begin{array}{l} {\bar{Y}}_{t}(0.5):\;=\frac{\left(Y_{t-1}(0.5)+Y_{t-2}(0.5)+Y_{t-3}(0.5)\right)}{3} \end{array}$$

The interest on non-maturing deposits is less than the interest on term deposits because non-maturing deposits are more liquid than term deposits. The time *t* interest payment on a non-maturing deposit (analogous for term deposits) with nominal 1 is then given by


(12) $$\begin{array}{l} I_{t}^{D}=e^{u_{t}^{D}/12}-1. \end{array}$$

Interest payments are assumed to be reinvested rather than paid out. The reinvestment of interest payments is treated in the same way as the reinvestment of maturing deposits. Deposits are the equivalent of loans on the right-hand side of the balance sheet as they can be seen as short positions in loans. The reinvestment of interest on deposits introduces an asymmetry between these two items, as interest on loans is assumed to be paid out.

#### 2.2.4. Decision-independent cash flow

All other costs that impact the bank's income are summarized as personnel and material costs. These have to be paid at each time step. Material costs are assumed to be the same for each time step, whereas personnel costs grow by 2% annually. The total costs paid at time *t* are denoted by *c*_*t*_ ∈ ℝ. Cash flows resulting from changes in loans and deposits, interest received on loans *r*_*t*_, and costs are independent of model decisions. Hence, the aggregated cash flow


(13) $$\begin{array}{l} CF_{t}:=\pi^{\left(1\right)}(R_{t})-∥{\tilde{R}}_{t}{∥}_{1}+r_{t}+∥{\tilde{S}}_{t}{∥}_{1}-\pi^{\left(1\right)}(S_{t})-c_{t} \end{array}$$

Can be calculated outside of the training loop.

### 2.3. Transition of decision-dependent variables

This section describes the evolution of model variables that depend on the investment and financing decisions made. This includes the value of the bank's equity at the final model step, which determines the reward (loss) assigned to a given set of actions. Consequently, the decision-dependent model variables have to be recalculated during the training to optimize the actions. The transitions of model variables in this section follow logically from fundamental relationships of *double-entry accounting*. The decision-dependent variables are the cash position, the investment portfolio, the financing portfolio, and consequently, the bank's assets, liabilities, and equities. Their transition can be split into the following iterative scheme: in each period, the balance sheet is *rolled forward*, investment and financing decisions are made, and the balance sheet gets *restructured* based on those decisions.

#### 2.3.1. Income statement

On the one hand, the *balance sheet* is always with respect to a snapshot in time and can be interpreted as a state variable. On the other hand, the *income statement* is always with respect to a certain time period and builds the bridge from the initial to the final balance sheet of that period. While all revenues and most costs result from cash flows of the balance sheet items listed earlier, operational costs such as personnel and material costs have to be accounted for in each model step. Furthermore, profit distributions are made annually to shareholders. Thus, it is essential to monitor equity over time. For our purpose, we do not need to break down the profit & losses (P&L) into explanatory components such as *net interest income, depreciations*, and *operational costs*. Instead, we simply track the gross and net P&L before and after *dividends*, respectively, on an aggregated basis; see later.

#### 2.3.2. Roll-forward step

At the beginning of each period, the cash flows associated with all balance sheet positions are realized. This step does not occur in period *t* = 0, implying that the initial balance sheet is given with no outstanding settlements. Recall that the cash flow resulting from maturing and newly issued loans and deposits, interest received on loans, and other costs has already been computed as the quantity *CF*_*t*_ outside of the training loop; see Equation (13). Furthermore, cash flows resulting from coupon and nominal payments in both the investment and borrowing bonds are realized. While the cash position is assumed not to earn positive interest, the bank might have to pay interest on its cash: in times when the short end of the yield curve is negative, the bank is granted a maximal allowance to deposit cash at the central bank which is exempted from negative interest. This amount is limited based on the minimum reserves MR of the bank; see Equation (24b) in the following for the exact terms. If the bank exceeds this limit in cash, it has to pay the market interest rate at the short end of the yield curve (i.e., for the maturity Δ*t*). This mechanism is modeled by charging the bank a cash penalty *cp*_*t*_ that corresponds to the negative interest the bank has to pay, namely


(14) $$\begin{array}{l} cp_{t}:\;={\left(C_{t}-30\times \text{MR}\right)}^{+}\left(\min\left\{\frac{1}{D_{t}(\Delta t)},1\right\}-1\right), \end{array}$$

which, due to its dependence on *C*_*t*_, is decision-dependent. Thus, during the roll-forward step, all cash flows together result in the cash update


(15) $$\begin{array}{l} C_{t+\Delta t}^{\text{pre}}=C_{t}+{\text{CF}}_{t}+\pi^{\left(1\right)}\left(B_{t}\right)-\pi^{\left(1\right)}\left(K_{t}\right)-{\text{cp}}_{t}, \end{array}$$

where *pre* indicates that $C_{t+\Delta t}^{\text{pre}}$ is not yet the cash at the end of period *t*+Δ*t*, but rather an intermediate quantity as it has not been updated yet by the bond transactions initiated at time *t*+Δ*t*.

As cash flows are realized, balance sheet positions have to be updated. Loans and deposit portfolios evolve as discussed in Section 2.2. Investment and borrowing bond portfolios also have to be rolled forward: the due amounts (i.e., the first entry in the vectors *B*_*t*_ and *K*_*t*_) are removed and all other payoffs are moved forward in time (entries in vectors are shifted up by one position), restoring the interpretation that the *k*^*th*^ entry of the vector *B*_*t*+Δ*t*_ represents cash flows being settled in period *t*+(1+*k*)Δ*t*. Finally, all cash flows need to be reevaluated under the prevailing yield curve at time *t*+Δ*t*. Therefore, the state of the balance sheet positions after the roll-forward step is thus given by


(16a) $$\begin{array}{l} B_{t+\Delta t}^{\text{pre}}=UB_{t}, \end{array}$$

(16b) $$\begin{array}{l} K_{t+\Delta t}^{\text{pre}}=UK_{t}, \end{array}$$

(16c) $$\begin{array}{l} V\left(B_{t+\Delta t}^{\text{pre}}\right)=\langle D_{t+\Delta t},B_{t+\Delta t}^{\text{pre}}\rangle , \end{array}$$

(16d) $$\begin{array}{l} V\left(K_{t+\Delta t}^{\text{pre}}\right)=\langle D_{t+\Delta t},K_{t+\Delta t}^{\text{pre}}\rangle , \end{array}$$

(16e) $$\begin{array}{l} A_{t+\Delta t}^{\text{pre}}=C_{t+\Delta t}^{\text{pre}}+V\left(B_{t+\Delta t}^{\text{pre}}\right)+V\left(R_{t+\Delta t}\right), \end{array}$$

(16f) $$\begin{array}{l} L_{t+\Delta t}^{\text{pre}}=V\left(S_{t+\Delta t}\right)+V\left(K_{t+\Delta t}^{\text{pre}}\right), \end{array}$$

(16g) $$\begin{array}{l} E_{t+\Delta t}^{\text{pre}}=A_{t+\Delta t}^{\text{pre}}-L_{t+\Delta t}^{\text{pre}}. \end{array}$$

#### 2.3.3. Restructure step

With the roll-forward of the balance sheet, the new period *t*+Δ*t* has now started, and the bank can make its investment decisions $a_{t+\Delta t}^{B}$ and financing decisions $a_{t+\Delta t}^{K}$. These decisions could be the result of any policy the bank wants to pursue, including one that directly parametrizes the actions with neural networks, i.e., the Deep ALM approach as presented in Section 3.2. The restructure step updates the balance sheet according to the investment and financing decisions made. This involves updating the bond portfolios by adding the cash flows of the newly bought and issued bonds to the existing portfolios at the correct maturities, i.e.,


(17a) $$\begin{array}{l} B_{t+\Delta t}=B_{t+\Delta t}^{\text{pre}}+\overset{b^{B}}{\underset{i=1}{\sum }}\pi^{\left(i\right)}\left(a_{t+\Delta t}^{B}\right)Z_{t}^{B,i}, \end{array}$$

(17b) $$\begin{array}{l} K_{t+\Delta t}=K_{t+\Delta t}^{\text{pre}}+\overset{b^{K}}{\underset{i=1}{\sum }}\pi^{\left(i\right)}\left(a_{t+\Delta t}^{K}\right)Z_{t}^{K,i}. \end{array}$$

Consistently, cash is updated as


(18) $$\begin{array}{l} C_{t+\Delta t}=C_{t+\Delta t}^{\text{pre}}-V\left(Z_{t}^{B,i}\right)+V\left(Z_{t}^{K,i}\right). \end{array}$$

Bond transactions affect bank's equity since transaction costs need to be borne (in terms of a spread). This implies that the decision-making and restructuring steps are not income-neutral, and we generally have $E_{t+\Delta t}\neq E_{t+\Delta t}^{\text{pre}}$. The value of equity at the end of the period can be calculated as


(19a) $$\begin{array}{l} V\left(B_{t+\Delta t}\right)=\langle D_{t+\Delta t},B_{t+\Delta t}\rangle , \end{array}$$

(19b) $$\begin{array}{l} V\left(K_{t+\Delta t}\right)=\langle D_{t+\Delta t},K_{t+\Delta t}\rangle , \end{array}$$

(19c) $$\begin{array}{l} A_{t+\Delta t}=C_{t+\Delta t}+V\left(B_{t+\Delta t}\right)+V\left(R_{t+\Delta t}\right), \end{array}$$

(19d) $$\begin{array}{l} L_{t+\Delta t}=V\left(S_{t+\Delta t}\right)+V\left(K_{t+\Delta t}\right), \end{array}$$

(19e) $$\begin{array}{l} E_{t+\Delta t}=A_{t+\Delta t}-L_{t+\Delta t}. \end{array}$$

The last restructure step is conducted at time *T*−Δ*t*. It is followed by a terminal roll-forward, whose resulting equity component will be decisive in the optimization exercise.

#### 2.3.4. Annual closing step

At the end of each year, the bank distributes a dividend δ_*t*_ amounting to 50% of its profits over the present year. The distributed cash directly decreases the bank's equity. On the monthly time scale, this translates into performing every 12^*th*^ time steps an additional update


(20a) $$\begin{array}{l} \delta_{t}=\frac{{\left(E_{t}-E_{t-1}\right)}^{+}}{2}, \end{array}$$

(20b) $$\begin{array}{l} C_{t}^{\text{post}}=C_{t}-\delta_{t}, \end{array}$$

(20c) $$\begin{array}{l} E_{t}^{\text{post}}=E_{t}-\delta_{t}, \end{array}$$

where *t* ∈ (𝕋\ {*T*}) ∩ℕ and *post* indicates that these are the cash and equity values after the dividend has been paid out.

### 2.4. Constraints

The bank operates in a highly regulated environment that imposes constraints on the bank's decisions. We are seeking for optimized control when adhering to all rules. We already restricted the bank's behavior inherently *via* the assumptions that all bonds are held-to-maturity and that short sales are not allowed. In addition, we take into account five regulatory constraints inspired by Basel III (see Basel Committee on Banking Supervision 2011), whose compliance is controlled whenever the balance sheet has been restructured. The weights below were determined in close collaboration with the bank to reflect the real weighting based on a more detailed accounting basis as closely as possible.

#### 2.4.1. Leverage constraints

To limit the leverage of banks, the Basel III framework divides the bank's capital into different tiers and places leverage constraints on each tier of capital. For model tractability, we summarize these constraints into a single leverage constraint on the ratio between equity *E*_*t*_ and *risk-weighted assets* RWA_*t*_. The latter is a weighted sum of the bank's assets, where the weights reflect the risk associated with each class. The constraint is defined as


(21a) $$\begin{array}{l} \frac{E_{t}}{{\text{RWA}}_{t}}\overset{!}{\geq }17\%, \end{array}$$

(21b) $$\begin{array}{l} {\text{RWA}}_{t}\;:\;=10\%\times V(B_{t})+35\%\times V(R_{t}^{P})+V(R_{t}^{E}). \end{array}$$

##### 2.4.1.1. Liquidity constraints

As opposed to previous regulations, the introduction of the Basel III framework placed a significant focus on liquidity risks that became particularly apparent during the financial crisis in 2008. In our framework, liquidity risks are monitored by two ratios, the *liquidity coverage ratio* (LCR) and the *net stable funding ratio* (NSFR). The LCR ensures that the bank has enough liquidity to cover the net cash outflow during a 30*d* stress period, denoted by ${\text{NO}}_{t}^{30}$. This outflow is approximated as a linear combination of the outstanding deposits and financing. *High-quality liquid assets* (HQLA) are required to exceed the net outflows by a buffer of at least 5%. More precisely,


(22a) $$\begin{array}{l} {\text{LCR}}_{t}:=\frac{{\text{HQLA}}_{t}}{{\text{NO}}_{t}^{30}}\overset{!}{\geq }105\%, \end{array}$$

(22b) $$\begin{array}{l} {\text{HQLA}}_{t}:=71\%\times C_{t}+89\%\times V\left(B_{t}\right), \end{array}$$

(22c) $$\begin{array}{l} {\text{NO}}_{t}^{30}:\;=17.6\%\times V(S_{t}^{D})+13.0\%\times V(S_{t}^{F})+1.0\%\times V(K_{t}). \end{array}$$

The NSFR aims to enforce liquidity over a longer horizon. It considers the ratio between the *available stable funding* (ASF) and the *required stable funding* (RSF) of the balance sheet. Similarly,


(23a) $$\begin{array}{l} {\text{NSFR}}_{t}:=\frac{{\text{ASF}}_{t}}{{\text{RSF}}_{t}}\overset{!}{\geq }105\%, \end{array}$$

(23b) $$\begin{array}{l} {\text{ASF}}_{t}:\;=95\%\times V(S_{t}^{D})+90\%\times V(S_{t}^{F})+60\%\times V(K_{t})\\ \;+100\%\times E_{t}, \end{array}$$

(23c) $$\begin{array}{l} {\text{RSF}}_{t}:=12\%\times V\left(B_{t}\right)+71\%\times V\left(R_{t}\right). \end{array}$$

In addition to LCR and NSFR, Swiss banks have to hold a *minimum reserve* at the SNB. In times of negative interest rates, the SNB demands higher reserves than usual. We frame this constraint *via* the *cash to minimum reserve ratio* (CMR) as


(24a) $$\begin{array}{l} {\text{CMR}}_{t}:=\frac{C_{t}}{{\text{MR}}_{t}}\overset{!}{\geq }100\%, \end{array}$$

(24b) $$\begin{array}{l} {\text{MR}}_{t}:=2.5\%\times (V(S_{t}^{D})+V(S_{t}^{F})(1-80\%\times 1_{\left\{Y_{t}\left(1/12\right)\geq 0\right\}})). \end{array}$$

##### 2.4.1.2. Interest rate sensitivity

The final constraint that is motivated from a regulatory perspective restricts the interest rate risk. To this end, one calculates the sensitivity of the bank's equity toward a parallel shift of the yield curve by ±100 bps. Let ${\tilde{E}}_{t}$ denote the residual equity if all other balance sheet items are reevaluated under the discount factors implied by the shifted yield curve. It is imposed that


(25) $$\begin{array}{l} {\text{IRS}}_{t}:=\frac{\left|{\tilde{E}}_{t}-E_{t}\right|}{E_{t}}\overset{!}{\leq }8.5\%. \end{array}$$

##### 2.4.1.3. Minimum annual return

Finally, we impose a lower bound on the annual revenue, which the bank is not supposed to undercut. It is motivated by preventing losses under any circumstances. The profit ought to exceed at least the basic operational cost plus an additional buffer of *m*CHF 6. Formulated in terms of the *excess yearly return-on-equity* (EYR), it reads


(26) $$\begin{array}{l} {\text{EYR}}_{t}:=\frac{\left(E_{t}-E_{t-1}\right)-6}{E_{t-1}}\overset{!}{\geq }0. \end{array}$$

This constraint is calculated on an annual basis during the annual closing step for *t* ∈ (𝕋\ {*T*}) ∩ℕ.

### 2.5. Objective

Formulating reward functions for real-world reinforcement learning applications is challenging, since one has to capture human preferences on the policy and its outcomes *via* a single number. ALM involves many stakeholders that have detailed and potentially different preferences on the ALM policy and the resulting evolution of balance sheet positions. Even the fundamental goal of ALM is ambiguous because the bank must trade off profitability versus hedging; see Spillmann et al. (2019, Chapter 2). As profits are recognized in equity, we act as if preferences in the ALM problem could actually be reduced to characteristics of the bank's equity distribution at the horizon *T*. The prerequisite that constraints should not be violated is additionally incorporated into the loss signal.

The assumption of solely focusing on the value of the bank's equity at time *T* might not truly capture preferences in this setting. Indeed, not all paths with the same final equity value *E*_*T*_ are valued equally from a practical perspective. Banks prefer their equity to be steadily increasing along its path to time *T* and are concerned with its maximum drawdown. This path preference is to some extent accounted for in the constraints; Equation (26) implies that equity paths with elevated drawdowns feature a higher loss provided that the minimum annual return has ever been violated at all. Otherwise, two equity paths will be evaluated as indifferent if they have the same final equity value. A possible remedy could entail to replace the single reward with compounded rewards based on, e.g., *E*_1_, *E*_2_, …, *E*_*T*_. We restrict our analysis to loss functions based only on *E*_*T*_ because even rewarding intermediate equity values does not entirely solve the more pressing issue of neglecting how well the bank will do after time *T*. Ignoring long-term success is not in alignment with true preferences as the bank will not be liquidated after time *T* but is a *going concern*. Ideally, this should not be problematic as all balance sheet items are valued fairly. If the cash flow structure of balance sheet positions is determined to be suboptimal for the bank after time *T*, it could simply be restructured without decreasing the bank's equity. In the presence of market frictions and short-selling constraints, it becomes questionable whether restructuring at negligible cost is possible. The experiments presented below indicate that the time horizon *T* has an impact on the learnt strategies. Furthermore, the bank seeks to avoid significant restructuring within short time periods.

The going concern principle motivates modeling the problem as an infinite decision problem, in which discounted rewards are issued periodically. DSC, as presented earlier, is not well suited for a problem with an infinite time horizon. We would require a different type of algorithm. Therefore, we restrict our formulation of the ALM problem to a finite time horizon *T*. If cutting off the problem leads to degenerate behavior toward the end of the model period, increasing the horizon *T* might make it less relevant: as long as there is enough time between today and time *T*, current actions might be unrelated to this behavior, and thus still be useful. We will investigate this issue later by comparing strategies for different model horizons *T*.

#### 2.5.1. Constant relative risk aversion

If preferences are rational^11^, maximizing preferences on the distribution of *E*_*T*_ becomes equivalent to maximizing the *expected utility* 𝔼[*u*(*E*_*T*_)], where the so-called *Bernoulli utility* *u*(*x*):ℝ → ℝ assigns a real value to a given realization *x* of *E*_*T*_. The assumption that investors are *risk averse* translates into the requirement that *u* is concave and non-decreasing. Since the underlying preference structure of the bank's shareholders is elusive, it is unclear what Bernoulli utility *u* describes the risk appetite most accurately. This problem is commonly approached by restricting *u* to be from a specific class of utility functions that are characterized by a small number of parameters. This includes the class of utility functions with *constant relative risk aversion* (CRRA), where *u* is of the form


(27) $$\begin{array}{l} u\left(x;\gamma \right)=\{\begin{array}{l} \frac{x^{1-\gamma }-1}{1-\gamma }\text{ if }\gamma \neq 1,\\ \log x\text{ if }\gamma =1. \end{array} \end{array}$$

As indicated by the name, relative risk aversion, defined by −*xu*″(*x*)/*u*′(*x*), equals γ for all *x* > 0. For DSC, the parameter γ is reverse engineered such that the terminal equity distribution of the learnt strategies is balanced. Because the ALM problem is framed as a minimization problem, we define the utility loss component ℓ^u^ as the negative utility of the equity ratio, i.e.,


(28) $$\begin{array}{l} \ell^{\text{u}}\left(E_{T}|E_{0};\gamma \right):=-u\left(\frac{{\left(E_{T}\right)}^{+}+\varepsilon }{E_{0}};\gamma \right); \end{array}$$

The equity ratio is floored at 0 < ε/*E*_0_ ≪ 1 to ensure that the CRRA utility remains well defined.

#### 2.5.2. Target return

Alternatively to the formulation as a utility maximization problem, Krabichler and Teichmann (2020) suggest framing the ALM problem as a hedging problem. Given an annual return target μ, this approach aims at minimizing the difference between the bank's final equity and the implied target value. This target loss is defined as


(29) $$\begin{array}{l} \ell^{\text{t}}\left(E_{T}|E_{0};\mu \right):={\left(E_{T}-{\left(1+\mu \right)}^{T}E_{0}\right)}^{2}. \end{array}$$

Economically, this loss function encodes a preference for adequate risk-adjusted returns. It has the advantage that the hyperparameter μ is easily interpretable as opposed to the abstract notion of the risk aversion coefficient.

#### 2.5.3. Penalties

The bank aims to maximize investor utility while sticking to several constraints. We encode this in the loss function by penalizing any violation of one of the six constrained quantities from Section 2.4. Denoting by $x_{t}^{i}$ the constrained quantity (e.g., LCR_*t*_) at time *t* and by β^*i*^ the bound corresponding to the constraint (e.g., 105% for LCR), the extent of the *i*^*th*^ breach at time *t* is calculated as


(30) $$\begin{array}{l} P_{t}^{i}:=\{\begin{array}{l} (1+{\left(x_{t}^{i}-\beta^{i}\right)}^{+}{)}^{2}-1\text{ if }i\in \left\{1,2,3,4,6\right\},\\ (1+{\left(\beta^{i}-x_{t}^{i}\right)}^{+}{)}^{2}-1\text{ if }i=5, \end{array} \end{array}$$

where *i* = 5 in the order of Section 2.4 corresponds to the interest rate sensitivity constraint. Taking the square of the violations encodes the preference that large violations are “more than linearly” worse than small violations. The intuition is that slight violations of a specific constraint are bad for the bank, while significant violations are detrimental.

The accumulated penalty *p* is defined as the weighted sum of all violations $P_{t}^{i}$. It is used to calculate the loss component for constraint violations


(31a) $$\begin{array}{l} \ell^{\text{p}}\left(p\right):={\left(1+p\right)}^{2}-1, \end{array}$$

(31b) $$\begin{array}{l} p:=\overset{6}{\underset{i=1}{\sum }}\sigma_{i}\overset{T-1}{\underset{t=0}{\sum }}P_{i}\left(x_{t}^{i}\right). \end{array}$$

The penalty is squared again, implying that large violations over the entire model period are “more than linearly” worse than small violations. The weights σ_*i*_ can be chosen to adjust for different magnitudes of the constrained quantities. Moreover, one can use these weights to encode preferences over the relative importance of different constraints. For instance, the weight of the penalty for violations of the minimum return is relatively small as this constraint is less binding than the regulatory constraints.^12^

#### 2.5.4. Loss

Finally, the loss associated with a single path *i* is given as a weighted sum of the utility loss and the penalty loss, i.e.,


(32) $$\begin{array}{l} \ell \left(E_{T},p|E_{0};\gamma ,\lambda \right)=\ell^{\text{u}}\left(E_{T}|E_{0};\gamma \right)+\lambda \ell^{\text{p}}\left(p\right), \end{array}$$

where λ > 0 determines the impact of the penalty on the total loss. The ALM problem is given by


(33) $$\begin{array}{l} \underset{{\left\{a_{t}\right\}}_{t\in \text{T}\backslash \left\{T\right\}}}{\min}\text{E}[\ell \left(E_{T},p|E_{0};\gamma ,\lambda \right)], \end{array}$$

where *E*_*T*_ and *p* result from the transition dynamics outlined in this section. Calculating the loss concludes the forward computations in the ALM framework. Algorithm 1 provides an overview in which order the presented steps are executed to obtain the final loss signal.

**Algorithm 1:**
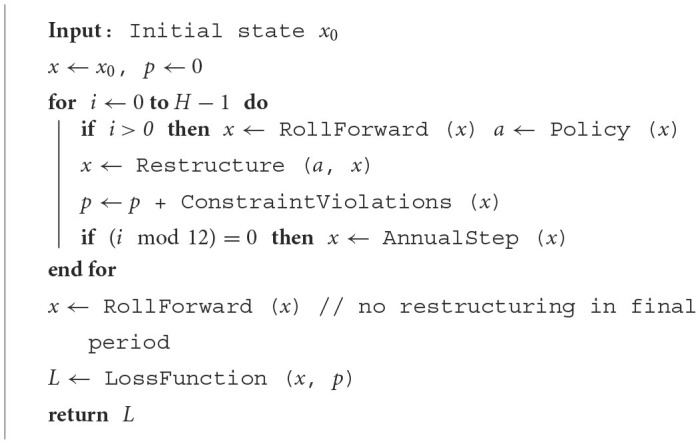
ALM.

### 2.6. Swaps

This section describes a model refinement by additionally incorporating plain-vanilla *interest rate swaps* into the decision process. Interest rate swaps are contracts between two counterparties that exchange floating payments for fixed payments. At predetermined times, one party (*payer*) pays a fixed payment and receives a floating payment, and the other party (*receiver*) receives the fixed payment and makes the floating payment. The fixed payments are called fixed as their amount is determined when the contract is entered into. The floating payments are determined throughout the duration of the contract based on a floating rate (e.g., LIBOR, up until recently, and compounded overnight rates). A brief and relevant introduction to swaps can be found in Filipović (2009, Chapter 1).

The basic ALM framework presented above is limited by the assumption that the bank can only invest in bonds and issue bonds. This neglects the bank's ability to control its interest rate exposure *via* swaps. In a second step, we extend the ALM framework by the introduction of interest rate swaps. Each period, the bank can additionally enter into *s* = 6 payer and receiver swaps, respectively, with maturities 5*y*–10*y*. Similarly to bonds, swaps cannot be sold in the secondary market and must be held until expiration. Swap payments occur on an annual basis starting exactly 1 year after issuance. The floating payments are determined 1 year before their payment and are given by the simple 1*y* spot rate prevailing at that time. The fixed payments are chosen such that the initial value of the swap is zero. The bank has to pay an annual spread of κ_*S*_ = 0.02% on both payer and receiver swaps. In absolute terms, the spread on swaps is smaller than the spread on bonds.

Recall that we keep track of bond positions by simply adding the entire cash flow of a bought or issued bond to the aggregated cash flow of the bond portfolio. This mechanism does not work for swaps as the cash flows of the floating leg are not known at issuance and are scenario-dependent. Instead, the position in each swap has to be kept separately. Computationally, this is done by keeping track of the holding portfolios $h_{t}^{pay}\in {\mathbb{R}}_{\geq 0}^{s\left(H-1\right)}$ and $h_{t}^{rec}\in {\mathbb{R}}_{\geq 0}^{s\left(H-1\right)}$ that denote the number of payer and receiver swaps, respectively, that are owned at a given time *t* ∈ 𝕋. The first dimension of $h_{t}^{pay}$ is the number of payer swaps that exist over the entire model horizon. Hence, each entry of the $h_{t}^{pay}$ denotes the volume with which a specific payer swap has been entered into at time *t*. The holding portfolios are initialized with zeros at all entries, i.e., we assume that there are no legacy swaps. At any time step *t*, the bank can then decide on the number of new payer swaps $a_{t}^{pay}\in {\mathbb{R}}_{\geq 0}^{s}$ and new receiver swaps $a_{t}^{rec}\in {\mathbb{R}}_{\geq 0}^{s}$ it wants to enter into. In the restructuring step, $a_{t}^{pay}$ ($a_{t}^{rec}$) is added to $h_{t}^{pay}$ ($h_{t}^{rec}$) at the correct indices.^13^ Also note that $a_{t}^{pay}$ and $a_{t}^{rec}$ have to be included in the control *a*_*t*_. In the extended setting, *a*_*t*_ is therefore of dimension 2(*b*+*s*) and a concatenation of $a_{t}^{B}$, $a_{t}^{K}$, $a_{t}^{pay}$, and $a_{t}^{rec}$.

The change in cash within the roll-forward step has to be adjusted to account for payoffs originating from swaps. If a swap *i* ∈ {1, 2, …, *s*(*H*−1)} has an exchange of cash flows in period *t* and the fixed payoff is given by *k*_*i*_, the net cash flow $\eta_{t}^{i}$ from the position in this swap is given by


(34a) $$\begin{array}{l} \eta_{t}^{{pay}_{i}}:=h_{t}^{{pay}_{i}}(\left(\frac{1}{D_{t-1}\left(1\right)}-1\right)-k_{i}-\kappa_{S}), \end{array}$$

(34b) $$\begin{array}{l} \eta_{t}^{{rec}_{i}}:=h_{t}^{{rec}_{i}}(-\left(\frac{1}{D_{t-1}\left(1\right)}-1\right)+k_{i}-\kappa_{S}) \end{array}$$

depending on whether we are dealing with a payer or receiver swap. The cash flows $\eta_{t}^{{pay}_{i}}$, $\eta_{t}^{{rec}_{i}}$ from all swaps *i* ∈ {1, 2, …, *s*(*H* − 1)} have to be added to the cash update in Equation (15). While the initial value of any swap contracts is zero (with the exception of a spread), swap positions have to be reevaluated in every roll-forward (Equation 16) and restructuring step (Equation 19). The fixed leg, the floating leg, and the associated spreads of each swap are valued using standard techniques involving the *forward rate curve*. The net value of all swap positions is considered to be an asset or liability for the bank if it is positive or negative, respectively. To calculate the required replacement values, we define the net swap assets *N*^*A*^ and net swap liabilities *N*^*L*^ as


(35a) $$\begin{array}{l} N^{A}:={\left(V\left(h_{t}^{pay}\right)+V\left(h_{t}^{rec}\right)\right)}^{+} \end{array}$$

(35b) $$\begin{array}{l} N^{L}:={\left(V\left(h_{t}^{pay}\right)+V\left(h_{t}^{rec}\right)\right)}^{-} \end{array}$$

and add *N*^*A*^ to the asset calculation and *N*^*B*^ to the liability calculation in the valuation step (Equation 16) and restructuring step (Equation 19), respectively.

We assume that the general calculation of constraints from Section 2.4 does not need to be adjusted in the extended setting. In particular, the impact of swaps on LCR, NSFR, and RWA is negligible. Still, the inclusion of swaps in the balance sheet impacts the leverage constraint on the ratio *E*_*t*_/RWA_*t*_. Furthermore, the interest rate sensitivity constraint is of course significantly impacted by the inclusion of swaps. The value of the swap portfolio impacts both the value of equity *E*_*t*_ and the value of equity under the shifted yield curve ${\tilde{E}}_{t}$. In the presence of swaps, this constraint becomes particularly important as the model could otherwise enter into positions with large exposures to interest rate risk.

While most balance sheet constraints remain unchanged, the number of payer swaps ($a_{t}^{pay}$) and receiver swaps ($a_{t}^{rec}$) that the bank can enter into in each period is constrained. Next to the solely computational requirement that these must be non-negative^14^, we place the liquidity constraint that $∥a_{t}^{pay}{∥}_{1}\leq 100$ and $∥a_{t}^{rec}{∥}_{1}\leq 100$. This implies that each month, the bank can only enter into payer and receiver swaps involving a notional amount up to *m*CHF 100. Finding counterparties for larger swap positions may not be easily possible in due course. Moreover, the total sum of outstanding payer and receiver swaps is required to be less than *m*CHF 3800 and *m*CHF 2800, respectively. More precisely, the constraints


(36a) $$\begin{array}{l} \overset{t}{\underset{s=0}{\sum^{\text{​}}}}∥a_{t}^{pay}{∥}_{1}\;\leq 3\;800, \end{array}$$

(36b) $$\begin{array}{l} \overset{t}{\underset{s=0}{\sum^{\text{​}}}}∥a_{t}^{rec}{∥}_{1}\;\leq 2\;800, \end{array}$$

must be satisfied for all *t* ∈ 𝕋\{*T*}. This is a simplification of a typical requirement from *hedge accounting*. The volume of payer and receiver swaps should not exceed the volume of unhedged assets and liabilities, respectively, at a given maturity. Correspondingly, swaps are intended to hedge outstanding interest rate risk, in contrast to taking on interest rate risk. The upper bounds were provided by the bank. They represent approximatively the volume of unhedged assets and liabilities that have maturities between 5*y* and 10*y*. While a dynamic recalculation of such limits would be more precise, we suspect that it should not impact results heavily, considering that loans and deposits evolve almost deterministically.

### 2.7. Term structure models

All approaches make use of a Monte Carlo approximation of the expected loss (Equation 33). This requires simulating a set of scenarios for the evolution of the yield curve, as discussed in Section 2.7. In principle, the approaches presented later can be applied to any set of yield curve scenarios. This general applicability does not mean that the “performance” of the different approaches does not differ based on the choice of simulated yield curves. Indeed, the contrary is the case: our experiments demonstrate how the yield curve simulator induces a bias in the model's decisions.

While treating the yield curve as a function *R*(*t*, ·):[*t*, ∞) → ℝ is mathematically convenient, prices are observed in practice for several types of bonds, but only for a limited number of maturities. For the Deep ALM framework, we need to model the yield curve at only *N* maturities. We therefore refer to the *N*-dimensional vector *Y* as the yield “curve”, where the *k*^*th*^ entry of *Y* is equal to *R*(*t, t*+*k*Δ*τ*) for a maturity step size Δτ. Apart from the last paragraph in Section 2.7.4, the following can be skipped by the knowledgeable reader.

#### 2.7.1. Simulation

We approach the ALM problem with a Monte Carlo-based deep learning method. The method uses a collection of scenarios to optimize the ALM decisions. Each scenario specifies the future development of variables that are relevant to the ALM problem. While some of those variables evolve deterministically, others are stochastic, i.e., differ between scenarios. The most important stochastic variable in ALM is the yield curve as it determines the rates at which the bank can lend and borrow money from both customers and investors. In fact, in our model of the ALM problem, the yield curve is the only source of randomness. Yield curve scenarios can be obtained by specifying a model for interest rate dynamics and then sampling from it. Ideally, this model satisfies the following criteria. First, it is financially reasonable to impose *absence of arbitrage* in the simulated bond market. Even statistical arbitrage is undesirable when using the simulation for training deep learning-based traders. They are likely to find and exploit risk-free profits that exist under the training distribution. But the simulated training distribution relies on an estimation of the mean returns of the traded assets (here bonds). If the estimation is flawed, trading strategies that were profitable under the simulated distribution are certainly not guaranteed to be so in practice; for a discussion in the related context of deep hedging, see Buehler et al. (2022a).

In the ALM framework developed in Section 2, the bank faces significant trading restrictions. This means that even if there exists arbitrage in the market, the bank might not be able to exploit this opportunity (at all, or at least on an arbitrarily large scale). This is pointed out similarly in the context of deep hedging by Buehler et al. (2019). First, the bank faces spreads when interacting with the market. Hence, an arbitrage opportunity can only occur when the payoff of this trading strategy net of the initial transaction costs is almost surely non-negative. Second, the bank is restricted to *long-only buy-and-hold-strategies* in its bond portfolios. This naturally restricts the set of trading strategies available to the bank.

When training a deep learning model on these paths, it is likely beneficial, if not necessary, to have a sufficiently rich class of yield curve scenarios; see also Reppen and Soner (2023). Having variability among scenarios helps the model explore the space of future attainable yield curves. This likely helps the performance of the deep learning model at inference on the real-world scenario. From an empirical perspective, it might be desirable to have a yield curve simulator that reproduces patterns observed in the past. Such stylized facts include that the yield curve tends to be shaped upwards, that short-maturity yields tend to fluctuate more than long-maturity yields, and that yield curve inversions usually happen when short-term rates are high; see Pedersen et al. (2016, p. 11).

The Deep ALM method splits the optimization into two parts: simulating a set of yield curve scenarios and then solving the optimization conditional on the simulated data. This means that irrespective of what model is chosen to simulate yield curves, the choice itself represents a source of *model risk* in the Deep ALM framework. This type of model risk is not unique to the Deep ALM framework but is present in many deep learning applications in quantitative finance; see Cohen et al. (2021).

#### 2.7.2. Svensson model

While for some maturities one might observe multiple prices in real-world fixed income markets as bonds are issued by different institutions, for other maturities they might not observe any bond prices at a given point in time. Hence, to obtain 'the' yield curve, some form of interpolation (or even extrapolation) is necessary. To this end, central banks such as the ECB or the SNB fit specific exponential-polynomial functions with a parsimonious parametrization to observed market yields. A popular choice is the model proposed by Svensson (1994), where the yield for a maturity *m*>0 is given by


(37) $$\begin{array}{r} R(t,t+m)=\beta_{0}+\beta_{1}\left(\frac{1-e^{\left(\frac{-m}{\tau_{1}}\right)}}{\frac{m}{\tau_{1}}}\right)\\ +\beta_{2}\left(\frac{1-e^{\left(\frac{-m}{\tau_{1}}\right)}}{\frac{m}{\tau_{1}}}-e^{\left(\frac{-m}{\tau_{1}}\right)}\right)\\ +\beta_{3}\left(\frac{1-e^{\left(\frac{-m}{\tau_{2}}\right)}}{\frac{m}{\tau_{2}}}-e^{\left(\frac{-m}{\tau_{2}}\right)}\right). \end{array}$$

The six parameters β_0_, β_1_, β_2_, β_3_, τ_1_, and τ_2_ are calibrated to fit observed market yields. Both ECB and SNB provide daily data of the fitted parameters from which historical yields for any maturity can be obtained. This is useful in the Deep ALM framework when calibrating a yield curve simulator to historical yield curves.

#### 2.7.3. Principal component analysis

From the previous analysis it is obvious that, at least for a fine grid size Δτ, the yield curve is a high-dimensional random vector with high dependencies between its elements. For modeling yield curve dynamics, it is natural to consider a lower-dimensional representation of the increments *via* a dimensionality reduction technique like *principal component analysis* (PCA); e.g., see Murphy (2012, Chapter 12). The yield curve dynamics can often be sufficiently described *via* its first three principal components; e.g., see Litterman and Scheinkman (1991). In this article, the approach to simulate yield curves is slightly different compared to classical PCA models. First, we apply a PCA on historical CHF forward curves and infer a deterministic, term-dependent volatility of a three-dimensional, risk-neutral HJM-type term structure. Second, PCA is utilized directly on simulated yield curves to obtain a low-dimensional representation of the yield curve as a feature for Deep ALM.

#### 2.7.4. Heath-Jarrow-Morton framework

Let the stochastic basis be a filtered probability space $\left(\Omega ,F,{\left(F_{t}\right)}_{t\geq 0},\mathbb{P}\right)$ in continuous time. Heath et al. (1992) proposed modeling the term structure of interest rates by specifying the stochastic evolution of the entire *instantaneous forward rate curve*. Under the no-arbitrage condition, these dynamics are fully specified by the volatility structure. A brief to the *HJM framework* can be found in Filipović (2009, Chapter 6).

Let α and σ be two stochastic processes, taking values in ℝ and ℝ^*d*^ that depend on two indices *t* and *T*, i.e., α = α(ω, *t, T*) and σ_*i*_ = σ_*i*_(ω, *t, T*) for all *i* = 1, 2, …, *d*. The forward rate process {*f*(*t, T*)}_*t*≥0_ for 0 ≤ *t* ≤ *T* is assumed to follow the dynamics


(38) $$\begin{array}{l} df\left(t,T\right)=\alpha \left(t,T\right)dt+\sigma \left(t,T\right)dW\left(t\right), \end{array}$$

where *W* denotes a *d*-dimensional Brownian motion under the objective measure ℙ. Equation (38) is well defined under some measurability and integrability assumptions; see Filipović (2009, Chapter 6) for further details. The initial forward curve *f*(0, *T*) is a model input and can be chosen to reflect the prevailing yield curve in the market. Heath et al. (1992) show that under an *equivalent local martingale measure* ℚ for the discounted bond price process, the forward rate dynamics 0 ≤ *t* ≤ *T* are given by


(39) $$\begin{array}{l} df\left(t,T\right)=\left(\sigma \left(t,T\right)\int_{t}^{T}\sigma {\left(t,s\right)}^{⊤}ds\right)dt+\sigma \left(t,T\right)dW^{*}\left(t\right), \end{array}$$

where W^*^ is a *d*-dimensional Brownian motion under ℚ. The HJM framework is very general and many classical interest rate models can be derived within it; see Brigo and Mercurio (2007, Chapter 5). Because the initial forward curve is a model input, HJM models match the initial term structure without any calibration. This is important in a practical application like ours and an advantage over simple short-rate models such as, e.g., Vašiček (1977). But there are also practical challenges associated with the large degree of freedom that the HJM framework offers. This includes the important choice of the volatility structure σ. For a general choice of σ, the short rate *r*(*t*) = *f*(*t, t*) is not Markovian, which, while undesirable for many practical applications, is not necessary for the Deep ALM framework. For the simple tenor-dependent volatility structure used in Section 2.7.5, the dynamics of the short rate actually are Markovian with respect to a finite-dimensional state; see Cheyette (2001).

For the remainder of this article, we make the simplifying assumption that the real-world measure and the risk-neutral measure coincide, i.e., ℙ = ℚ. This assumption could be relaxed by exogenously specifying the *market price of risk*. While this would not change the general ALM framework developed in the next sections, it would impact the learnt strategies and possibly lead to different interpretations.

#### 2.7.5. Tenor-dependent HJM model

Specifying a yield curve model that meets all the requirements from Section 2.7.1 is not trivial. Simple short rate models, such as Vašiček (1977), are not well suited as their few degrees of freedom have to be used for calibration to the initial yield curve. The HJM-type model that we use to simulate yield curves assumes a simple structure of the instantaneous volatilities. These are assumed to be constant over time and tenor-dependent. The method matches the initial yield curve inherently and generates a variety of shapes.

In the following, the forward curve refers to the random *N*-dimensional vector *F*_*t*_ instead of the function *f*(*t, T*). The *j*^*th*^ entry of *F*_*t*_ is equal to *f*(*t, t*+*j*Δ*τ*) for some discretization size Δτ. Similarly, $A_{t}\in {\mathbb{R}}^{N}$ denotes the vectorized version of α(*t, T*) and $V_{t}\in {\mathbb{R}}^{N\times d}$ denotes the vectorized version of σ(*t, T*). Note that the latter is a matrix as σ(*t, T*) already is a *d*-dimensional vector. Evaluating (38) at all the tenors of *F*_*t*_ yields the *N*-dimensional stochastic differential equation


(40) $$\begin{array}{l} dF_{t}=A_{t}dt+V_{t}dW_{t}, \end{array}$$

describing the dynamics of the forward curve vector. In this form, the instantaneous volatility structure is captured by the matrix *V*_*t*_, which fully determines the drift *A*_*t*_ under the risk-neutral measure; see Equation (39). For modeling purposes, one has to specify the dynamics of {*V*_*t*_}_*t*≥0_. We use a particularly simple model where the instantaneous volatility structure is assumed to be constant over time, i.e., *V*_*t*_ ≡ *V*. This means that the forward curve is exposed to the *d*-dimensional shock *W*_*t*_ with a possibly different magnitude at each tenor. The *j*^*th*^ column vector of *V*, denoted *V*^(*j*)^, specifies the tenor-dependent exposure to $W_{t}^{j}$. The matrix *V* is fitted to historical data by decomposing the historical forward curve increments into its principal components. Moreover, this relies on the assumption ℙ = ℚ. The precise fitting method is as follows:

1. Estimate the covariance matrix $\hat{\Sigma }$ of weakly forward curve changes {Δ*F*_*t*_}_*t* = 0, 1, …, *T*_ and annualize it by multiplying $\hat{\Sigma }$ by 52.
2. Apply an eigendecomposition on the scaled, estimated covariance matrix, i.e., $\hat{\Sigma }=Q\Lambda Q^{-1}$, where the columns of *Q* ∈ ℝ^*N*×*N*^ are the eigenvectors of $\hat{\Sigma }$ and Λ ∈ ℝ^*N*×*N*^ is the diagonal matrix containing the eigenvalues of $\hat{\Sigma }$.
3. Keep the first *d* = 3 eigenvectors and scale them by their eigenvalues, i.e., ${\tilde{V}}^{\left(j\right)}:=\lambda_{j}Q^{\left(j\right)}$ for *j* = 1, 2, …, *d*.
4. To regularize the estimate, approximate the vectors ${\tilde{V}}^{\left(j\right)}$ as polynomial functions of the tenors. The degree of the polynomials is a modeling choice; we use cubic polynomials.
5. Set the *j*^*th*^ column vector of *V* equal to the *j*^*th*^ fitted polynomial evaluated at the relevant tenors.

We model σ(*t, T*) as a *d*-dimensional vector where the entries are polynomials as functions of the tenor *T*−*t*. Following the HJM approach, the risk-neutral drift *A*_*t*_ ≡ *A* is given by the drift in Equation (39) and can be approximated using the trapezoidal rule. Future forward curve paths can be simulated *via*


(41) $$\begin{array}{l} F_{t+\Delta t}=F_{t}+\left(A+\frac{\partial F_{t}}{\partial \tau }\right)\Delta t+V\left(W_{t+\Delta t}-W_{t}\right), \end{array}$$

where ∂_τ_*F*_*t*_ is approximated as the forward difference.^15^ Finally, the *k*^*th*^ entry of the vector of zero coupon bond prices *D*_*t*_ (*discount factors*) is calculated by the approximation from Glasserman (2003, Chapter 3)


(42) $$\begin{array}{l} D_{t}^{k}=\exp\left(-\underset{j\leq k}{\sum }F_{t}^{j}\Delta \tau \right), \end{array}$$

where the superscripts refer to the components of the respective vectors. We refer to this method as the *HJM-PCA approach*. The following hyperparameters are associated with it: the start and end date of the historical data used to fit the principal components, the time discretization (here *weekly*), the number of principal components fitted, and the degrees of the polynomials fitted to the principal components. See Table 2 for our concrete choices.

**Table 2:** Hyperparameters—There are many hyperparameters within the Deep ALM framework.

**Hyperparameter**	**Value**
**Yield curve simulation (HJM PCA)**	
Number of principal components	3
Degree of polynomials	3
Yield curve data period for PCA (data source: SNB)	01.01.2005 - 15.07.2022
**Neural network parameters**	
Dimension of hidden encoding layers	64
Dimension of encoding	32
Dimension of hidden layers	[512, 512, 256, 128]
Activation function	ELU
**Loss function parameters**	
Target parameters: [μ^*d*^, μ^*u*^], μ^eval^	[2%, 7%], ~4%^*^
Penalty coefficients: σ_*i*_ (LCR, NSFR, CMR, E/RWA, IRS, EYR)	[1.0, 0.2, 1.0, 2.5, 2.0, 0.002]
Penalty weight: [λ^*d*^, λ^*u*^], λ^eval^	[0.05, 25.0], 3.5
**Training parameters**	
Epochs^**^	100
Training scenarios^**^	40,000
Batch size	32
**Optimization parameters**	
Optimizer	RAdam
Learning rate	cyclic scheduler on [5e-4, 5e-3]
Gradient clip value	0.2

This table presents the most important ones, excluding the hyperparameters of the ALM simulation as presented in the text and the notation tables in the Appendix.

* Differs based on the setting. See Table 3 for the exact choices.

** The best models are fine-tuned on constantly resimulated paths.

**Figure 4:**
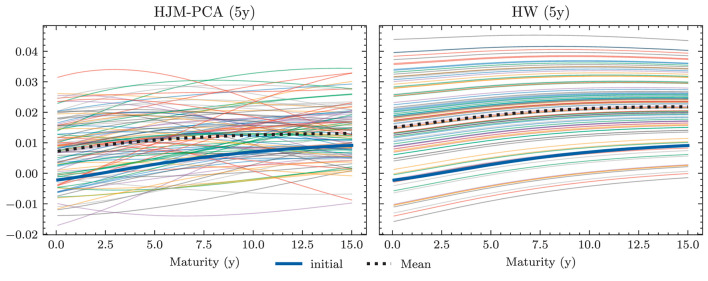
Simulated yield curves in 5*y*—The left-hand side shows a random sample of the terminal term structure simulated with HJM-PCA over a horizon of 5*y*. We encounter a rich family of different shapes. On the right-hand side, we see a random sample generated by a Hull-White-extended Vašiček model calibrated to the recent past; e.g., see Brigo and Mercurio (2007, Chapter 5). We chose the long-term mean time-dependent to match the initial yield curve and left the mean reverting rate as well as the instantaneous volatility constant. Regarding Deep ALM, the encountered diversity is not sufficient to get convincing results.

**Figure 5:**
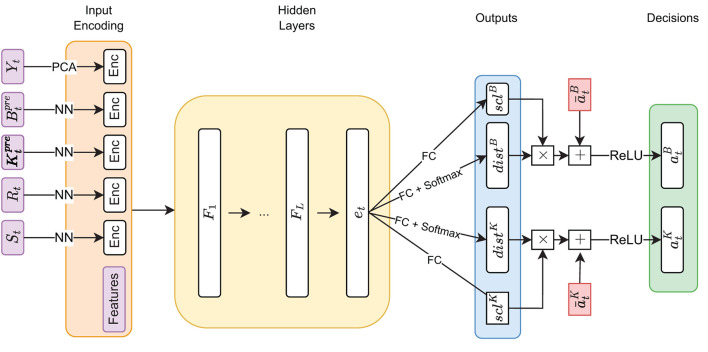
Decision network architecture—arrows annotated with “NN” denote a *forward pass* through a shallow neural network, the annotation “FC” denotes a *single fully connected* layer. The notations *scl* and *dist* are used to indicate the *scale* and *distribution* decisions made by the model.

Figure 4 shows the 1*m*-yields simulated using the HJM-PCA approach. Short-term rates are increasing in most scenarios which is roughly in line with expectations expressed by the bank on physical realizations of future yields. In more than 95% of the scenarios, the short-term yield stays above −1%. This is desirable for our application as many of the modeling assumptions in the ALM framework would be poor if yields were to fall to historically unprecedented negative levels. The left-hand side of Figure 5 shows entire yield curves attained using the HJM-PCA approach to simulate 5 years into the future. Most yield curves lie above the initial curve and there is a decent variety of yield curve shapes. In the mean, yield curves have a slightly positive slope that is smaller than that of the initial yield curve. According to the bank, this is a reasonable assumption as the yield curve has been artificially steep in times of negative interest rates. Overall, the simulated yield curves seem reasonable and suited for our application. While the HJM-PCA model is certainly not perfect, yield curve simulation is not the main focus of this article. Instead, the focus lies on optimizing an ALM policy conditional on a set of simulated yield curves. The striking feature of our Deep ALM approach is that one can easily substitute the HJM-PCA model by any other method for simulating yield curve movements.

## 3. Deep ALM

In this section, we present approaches for solving the ALM problem defined in Section 2: how should the investment and financing decisions (*a*_*t*_)_*t* ∈ 𝕋\{*T*}_ be made to minimize the expected loss (Equation 33)? We start by presenting simple benchmarks (Section 3.1) and then describe the full Deep ALM approach for tackling the basic ALM setting without swaps.

### 3.1. Benchmarks

Defining an algorithmic benchmark that mimics how banks make ALM decisions in practice is virtually impossible. These decisions are often made through a combination of simple models and expert judgment. Having a strategy that can easily be computed is of course one of the main motivations behind Deep ALM. For benchmarking purposes, we therefore define and optimize strategies endogenously in the ALM framework. The benchmarks were especially valuable during the development of the deep learning models because the benchmarks are much simpler to optimize.

#### 3.1.1. Equal allocation

A naive approach for choosing the investment and financing maturities is the 1/*N* strategy. Each period, an amount $∥a_{t}^{B}{∥}_{1}$ and $∥a_{t}^{k}{∥}_{1}$ is split equally among all investment and financing bonds, respectively. In the simplest case, this amount is the same in each period leading to the constant policies $a_{t}^{B}\equiv a^{B}$ and $a_{t}^{K}\equiv a^{K}$. Hence, this policy is characterized by only two parameters ∥*a*^*B*^∥_1_ and ∥*a*^*K*^∥_1_. Having to invest or borrow the same amount in each period is very restrictive. Instead, one can pursue a 1/*N* strategy where the scales $∥a_{t}^{B}{∥}_{1}$ and $∥a_{t}^{K}{∥}_{1}$ are time-dependent. This strategy has 2(*H*−1) parameters, as there are *H*−1 periods where decisions are made.

#### 3.1.2. Optimal constant allocation

A slightly more sophisticated benchmark strategy can be built by relaxing the 1/*N* assumption and choosing a potentially more optimal distribution over the investment and financing maturities instead. Such a benchmark strategy can be specified with different degrees of freedom. In the simplest case, where decisions are assumed to be constant over time, this strategy has *b*^*B*^+*b*^*K*^ parameters. When decisions are allowed to vary over time, the number of parameters increases to (*b*^*B*^ + *b*^*K*^) × (*H* − 1). When investment and financing maturities differ, as it is the case in our setup, equal allocation introduces an asymmetry in the duration of investment and financing decisions. In our case, where the maturities 3*m*, 1*y*, and 2*y* are only available for financing purposes, investments under the 1/*N* strategy have a higher duration than borrowings.

Each of these benchmarks makes the same decision in each scenario at a given model step *t* ∈ 𝕋\{*T*}. This is in contrast to the Deep ALM approach presented in Section 3.2, which tries to optimally adapt a given strategy to the current balance sheet structure and interest rate environment. The simplicity of the benchmark strategies makes them useful for more than just comparison purposes. Because they are easy to interpret, these strategies can provide valuable insights for ALM practitioners. The parameters of the benchmark strategies are optimized using gradient descent (see the optimizer in Table 2), i.e., in the same way that weights of neural networks are optimized in the Deep ALM approach. While this is straightforward within our ALM framework, it is already more complex than many prevalent tools in practice.

The benchmark strategies, where the scale of investments $∥a_{t}^{B}{∥}_{1}$ and borrowings $∥a_{t}^{K}{∥}_{1}$ is constant over time, perform poorly because legacy investments and borrowings are not equally distributed among maturities. This implies that there are periods where large tranches of investments and borrowings mature, and other periods where rarely any legacy positions mature. Considering this structure of the legacy portfolios, forcing a constant scale among investment and financing activities is undesirable. The default mechanism should instead be that maturing positions in the bond portfolios are rolled over. Consequently, we define the scale of investments in all benchmark strategies as follows:


(43) $$\begin{array}{l} ∥a_{t}^{B}{∥}_{1}=\pi^{\left(1\right)}(B_{t}^{\text{pre}})+\theta_{t}^{B}, \end{array}$$

where $\pi^{\left(1\right)}\left(B_{t}^{\text{pre}}\right)$ are the investments that mature next period and $\theta_{t}^{B}$ becomes the learnt parameter that may be shared over time. The scale of financing decisions $∥a_{t}^{K}{∥}_{1}$ is defined analogously with scale parameter $\theta_{t}^{K}$. The investment and borrowing scales are then multiplied with the learnt or specified distribution over the available maturities. Note that $a_{t}^{B}$ and $a_{t}^{B}$ are ensured to have no negative entries, i.e., adhere to the long-only constraint. For the analysis in Section 4.1.1, we restrict ourselves to comparing the following benchmarks:

– *BM*^*E*^: 1/*N* strategy with shared scale across time and two parameters
– *BM*^*C*^: strategy with learnt allocation that is shared across time and *b*^*B*^+*b*^*K*^ parameters
– *BM*^*D*^: dynamic strategy with learnt allocation, i.e., not shared over time, and (*b*^*B*^ + *b*^*K*^) × (*H* − 1) parameters

### 3.2. DSC for ALM

Deep ALM applies the key idea from DSC to the ALM problem. At each *t* ∈ *T*\{*T*}, the decision *a*_*t*_ is parametrized with a neural network $g^{\theta_{t}}:{\mathbb{R}}^{d}\to {\mathbb{R}}^{b^{B}+b^{K}}$, which we call the *decision network*. This means that the decision *a*_*t*_ is given by the *forward pass*


(44) $$\begin{array}{l} a_{t}=g^{\theta_{t}}\left(X_{t}\right), \end{array}$$

where $X_{t}:\Omega \to {\mathbb{R}}^{d}$ denotes the features passed to the neural network. These represent the relevant and observable information that the neural network needs to make qualified investment and financing decisions at time *t*. While this parametrization is conceptually simple, we find that to learn good strategies, details matter. What features are important? How should the architecture of $g^{\theta_{t}}$ look like? How can one make the optimization stable and robust? We discuss these questions in the following sections.

#### 3.2.1. Features

##### 3.2.1.1. Yield curve

By being the only source of randomness in the model, the currently prevailing yield curve *Y*_*t*_ is an important input feature. To reduce input dimensionality, we use PCA to project the high-dimensional yield curve into ℝ^3^. The low-dimensional representation of the yield curve is then used as an input feature. The PCA is performed before training on a subset of yield curves from the training data set. This gives similar results as using several (more than three) points on the yield curve as input features. If the yield curve dynamics used are non-Markovian, it might make sense to also provide some (compressed) form of the yield curve history to the model. Alternatively, one can let the model learn a compression of the yield curve history. This compression can then be passed from one decision network to the next and updated by the currently observable yield curve (in the same way that hidden states evolve in recurrent neural networks). When using the HJM-PCA model for simulation, neither approach improves model performance, which is expected as the yield curve dynamics are Markovian.

##### 3.2.1.2. Portfolios

The model needs to be aware of the cash flow structures of all balance sheet positions. Knowing the current investment and financing portfolios, $B_{t}^{\text{pre}}$ and $K_{t}^{\text{pre}}$ are essential, especially because they depend on previously made decisions. The loan portfolio *R*_*t*_ does not depend on previous decisions and differs only slightly between the different scenarios due to depreciation. The deposit portfolio *S*_*t*_ does not differ at all between scenarios as it is assumed to be deterministic. Still, these portfolios of course differ across time. Instead of forcing the model to remember the portfolios, *R*_*t*_ and *S*_*t*_ are provided as features, which is important when weights are shared; see later. Before passing the portfolios to the main network, we reduce their dimensionality. Each *N*-dimensional portfolio is mapped to lower-dimensional representation *via* a single fully connected layer, whereby different encoding layers for $B_{t}^{\text{pre}},K_{t}^{\text{pre}},R_{t},$ and *S*_*t*_ were used. Providing the entire high-dimensional portfolios to the encoding layers and using fully connected encoding layers have worked best in our experiments. Only providing portfolio duration or other lower-dimensional representations (e.g., *via* pooling) deteriorates performance. Replacing the linear encoding layer with other encoding architectures using convolutions or self-attention did not improve performance in our experiments. Similarly, training a single encoder on the stacked vector of all portfolios did not lead to better results.

##### 3.2.1.3. Relative size of balance sheet items

The features also include the following ratios that fully describe the aggregated balance sheet on an absolute and relative scale, namely


(45a) $$\begin{array}{l} X_{t}^{\text{size}}=A_{t}^{\text{pre}}/A_{0}, \end{array}$$

(45b) $$\begin{array}{l} X_{t}^{\text{lev}}=E_{t}^{\text{pre}}/A_{t}^{\text{pre}}, \end{array}$$

(45c) $$\begin{array}{l} X_{t}^{\text{liq}}=C_{t}^{\text{pre}}/A_{t}^{\text{pre}}, \end{array}$$

(45d) $$\begin{array}{l} X_{t}^{\text{inv}}=B_{t}^{\text{pre}}/A_{t}^{\text{pre}}, \end{array}$$

(45e) $$\begin{array}{l} X_{t}^{\text{fin}}=K_{t}^{\text{pre}}/A_{t}^{\text{pre}}. \end{array}$$

##### 3.2.1.4. Constraint features

To provide the model with recent information on its constraints across the network and time instances, we add the constrained quantities $x_{t-1}^{i}$ (e.g., LCR_*t*−1_), as calculated in the previous period^16^, as additional features. The interest rate sensitivity is added directly as a feature. For the other constrained variables, the difference between their value $x_{t-1}^{i}$ and the lower bound β^*i*^ is added as a feature.

#### 3.2.2. Architecture

**Figure 6:**
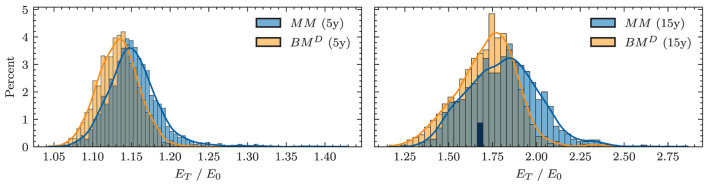
Equity ratio histogram—This chart depicts the final equity distribution of Deep ALM after 5*y* and 15*y*, respectively, compared to the most competitive benchmark strategy.

In this section, we describe the exact architecture of the neural network $g^{\theta_{t}}$ as illustrated in Figure 6. The main idea is to conceptually split different functions within the model. First, the model input is obtained by encoding the yield curve, encoding the portfolios, and calculating all other features. In concatenation, this input is passed through a feed-forward neural network; see Table 2 for the configuration. The output of this sub-network, the *d*′-dimensional final encoding *e*_*t*_ is then mapped to investment and financing decisions *via*


(46a) $$\begin{array}{l} a_{t}^{B}=[{\bar{a}}_{t}^{B}+g_{scl^{B}}^{t}\left(e_{t}\right)\times g_{dist^{B}}^{t}\left(e_{t}\right){]}^{+} \end{array}$$

(46b) $$\begin{array}{l} a_{t}^{K}=[{\bar{a}}_{t}^{K}+g_{scl^{K}}^{t}\left(e_{t}\right)\times g_{dist^{K}}^{t}\left(e_{t}\right){]}^{+}, \end{array}$$

where ${\bar{a}}_{t}^{B}$ and ${\bar{a}}_{t}^{K}$ are the investment and borrowing decisions of the pre-trained benchmark strategy *BM*^*D*^, $g_{scl^{B}}^{t}$ is a linear layer with no activation function and scalar output (the investment scale), and $g_{dist^{B}}^{t}\left(e_{t}\right)$ is a linear layer with softmax activation and *b*^*B*^-dimensional output (the investment distribution). The layers $g_{scl^{K}}^{t}$ and $g_{dist^{K}}^{t}$ are defined analogously.

This output mechanism is constructed for a couple of reasons. The pre-trained benchmark strategy *BM*^*D*^ is leveraged to reduce training time and to help with the scale of the model output. The output vectors $a_{t}^{B}$ and $a_{t}^{K}$ might reasonably have an *L*^1^-norm that lies in the thousands. This has the interpretation that total investments or borrowings exceed *m*CHF 1, 000 in a given period. Learning outputs of that scale directly is more challenging than learning only those investments or borrowings that are made in deviation from the benchmark.^17^ The output scale of the neural network thus matters at least in the sense that it makes finding good hyperparameters easier; see also van Hasselt et al. (2016). This further motivates splitting the excess decision into a scale decision and a distribution decision. To adhere to the long-only constraint, a ReLU layer is applied to the final investment and financing decisions. Next to the output scale, scaling the input portfolios $B_{t}^{\text{pre}}$, $K_{t}^{\text{pre}}$, *R*_*t*_, and *S*_*t*_, before passing them to the encoding layers, further improves the learning process. Simply dividing the portfolio positions by a factor of 100 works best in our experiments. Learning scaling parameters of the neural network's input or hidden state *via batch normalization* (Ioffe and Szegedy, 2015) did not improve (on the contrary, it rather deteriorated) the learning process.

#### 3.2.3. Optimization

As with many problems in reinforcement learning, the Deep ALM optimization is challenging and does not work well 'out of the box'. This is likely due to the problem having many local minima. Many decisions, such as whether to invest a given amount into a 10*y* or 11*y* bond, can have a negligible impact on the loss. On the other hand, if a change of parameters leads to a violation of constraints, the loss quickly explodes due to large gradients. Just scaling down the penalty weight λ or replacing the squared dependencies in the penalty calculation (Equations 30, 31) with linear dependencies does not solve this problem but simply leads to learning solutions with more violations of constraints. Another reason for the optimization difficulty is the recurrent structure of the problem because gradients are vanishing when backpropagated through time; see Hochreiter (1998). This is unsurprising due to the computational similarity to recurrent neural networks. To improve the learning process, several techniques from the deep learning literature are used.

As discussed in Section 2.5, it is unclear which loss function corresponds to underlying ALM preferences. Following Krabichler and Teichmann (2020), two alternatives were presented: a loss function based on the CRRA utility and a quadratic hedging criterion. Selecting a loss function and its parameters is an important and delicate matter, which can be tackled by monitoring alternative metrics (see Section 3.3) and analyzing the learnt strategies in detail. Irrespective of what loss function better encodes preferences, we find that the learning process seems to work better when using the target-based rather than the utility-based loss function. Despite both loss functions encoding different objectives, models trained on the target loss function often achieve equal (sometimes even higher) CRRA utility than models that were trained using CRRA utility. In terms of other metrics, such as the VaR of the final equity distribution, models trained on the target loss seem to perform better. All models are thus trained with the target loss function, while the CRRA utility is additionally used for model evaluation. The target loss function is applied with some adjustments. First, only downward deviations from the target return are penalized. Economically, it makes sense that higher returns should not be penalized. Practically, this leads to better performance in terms of other metrics. This asymmetric target loss ℓ^t^ on a single path with a given return target μ is therefore given by


(47) $$\begin{array}{l} \ell^{\text{t}}\left(E_{T}|E_{0};\mu \right)={\left[{\left(E_{T}-{\left(1+\mu \right)}^{T}E_{0}\right)}^{-}\right]}^{2}. \end{array}$$

Replacing the utility loss ℓ^u^ with this target loss ℓ^t^ in Equation (32) gives the total loss function used in our numerical experiments.

##### 3.2.3.1. Sampling loss function parameters

Using the target loss means having to choose an annualized return target μ. This choice is important because if the chosen target is too large, the model will take unreasonably high risks to match the target. If it is too small, the learnt policy will not be optimal in any practical or expected utility sense. Alleviating the impact of the target choice is another motivation for only penalizing downward deviations in the target loss. When using the symmetric target loss (29) in combination with an unambitious return target μ, we observe an undesirable strategy: the model maximizes equity during the first few periods, overshoots the target in many scenarios, and then decreases the bank's equity by taking unprofitable actions. Even with the asymmetric target loss, it is unclear how to choose the exact target μ. Instead of restricting the optimization to a single return target, a different target is sampled for each path during each epoch from a uniform distribution on the interval [μ^*l*^, μ^*u*^]. Initially, the motivation behind this approach was the following: we provide the sampled target as an additional feature to the neural network and optimize actions conditional on a given return goal. This approach of *upside-down reinforcement learning* (Schmidhuber, 2020; Srivastava et al., 2021), essentially translates the reinforcement learning problem into a supervised learning problem when viewing the obtained reward as the *prediction* and the target reward as the *label*. The idea of conditioning on a parameter of the loss function has also been successfully applied to similar problems in finance by conditioning on a risk aversion parameter; see Leal et al. (2021) and Murray et al. (2022).

Applying this technique of sampling the target μ and providing it as a feature to the neural network improves model performance across all relevant metrics. However, the learnt strategies do not differ significantly when varying μ at inference, i.e., there is no adaptive behavior in the sense of upside-down reinforcement learning. The target sampling may instead be interpreted in a probabilistic sense: the true target return μ that corresponds to the preferences of the bank's shareholders is unknown but assumed to be uniformly distributed on [μ^*l*^, μ^*u*^]. The improvement in performance may be due to increased exploration when the target is sampled. Furthermore, note that the mean of the uniform distribution on the chosen interval [μ^*l*^, μ^*u*^] in Table 2 is an ambitious return target in the sense that it lies significantly above the mean return that the best models achieve in the experiments; see Table 3.

**Table 3:** Main results—*MM* abbreviates *main model*, which represents the trained Deep ALM model.

**Model**	***BM* ^*E*^**	***BM* ^*C*^**	***BM* ^*D*^**	***MM***	***MM* ^*S*^**	***MM***	***BM* ^*E*^**	***BM* ^*C*^**	***BM* ^*D*^**	***MM***
**Horizon**	**5*y***	**5*y***	**5*y***	**5*y***	**5*y***	**15*y*|5*y***	**15*y***	**15*y***	**15*y***	**15*y***
**Loss statistics**										
$\bar{\ell }{\left(\lambda =3.5\right)}^{*}$	1.357	0.999	0.753	0.467	1.611	−	45.907	32.151	3.123	1.587
${\bar{\ell }}^{\text{t}}{\left(\mu =4.06\%\right)}^{**}$	1.428	0.997	0.824	0.558	1.719	45.290	10.829	10.625	3.059	1.712
${\bar{\ell }}^{\text{p}}$	0.011	0.032	0.012	0.003	0.002	0.007	10.066	6.193	0.067	0.015
${\text{ES}}_{0.95}\left(\ell^{\text{p}}\right)$	0.107	0.518	0.144	0.036	0.035	0.062	185.857	114.694	0.836	0.124
${\bar{\ell }}^{\text{u}}\left(\gamma =10.0\right)$	−0.064	−0.071	−0.074	−0.079	−0.084	−0.071	−0.106	−0.107	−0.110	−0.110
**Equity ratio statistics**										
$\bar{\text{ER}}$	1.107	1.124	1.133	1.153	1.173	1.128	1.518	1.514	1.716	1.812
σ_ER_	0.040	0.026	0.027	0.036	0.034	0.043	0.178	0.164	0.172	0.202
skew(ER)	−0.419	0.047	0.286	1.682	1.068	1.263	0.707	0.946	0.033	0.398
kurt(ER)	2.551	0.883	1.088	8.101	4.674	5.465	4.079	4.913	1.127	0.657
VaR_0.95_(ER)	−0.071	−0.043	−0.043	−0.049	−0.050	−0.064	−0.273	−0.242	−0.302	−0.320
ES_0.95_(ER)	−0.101	−0.055	−0.053	−0.060	−0.062	−0.082	−0.356	−0.308	−0.368	−0.368
**Annualized statistics**										
$\bar{\mu }$ (in %)	2.048	2.353	2.529	2.883	3.242	2.434	2.781	2.768	3.631	4.002
$\bar{\delta }$ (in %)	1.432	1.503	1.560	1.855	2.147	1.613	3.042	2.982	3.967	4.515

The superscript *S* indicates the inclusion of swaps; see Section 4.5. *BM* is short for *benchmark*. Recall that they assign constant weights regardless of the scenarios. *BM*^*E*^ and *BM*^*C*^ make the same decisions along the time axis. Regarding *BM*^*E*^, the distribution is fixed and only the scale is learnt. Both distribution and scale are trained in *BM*^*C*^. Finally, *BM*^*D*^ acquires adaptive weights along the time axis.

^*^ The mean loss is a linear combination of the losses reported later.

^**^ The first four listed models are evaluated with μ = 4.06%, model *MM*^*S*^ is evaluated with μ = 5.39%, and the 15*y* models are evaluated with μ = 4.00%. Hence, total and target loss should not be compared between these different categories.

It is also unclear how to choose the loss function weight λ that trades off the target objective and the penalty avoidance objective. If λ is too small, the model learns strategies that violate constraints in too many scenarios. If λ is too large, the target objective is effectively neglected and the performance in terms of the validation metrics decreases. Again, having to make a single choice (at least for training) is avoided by sampling a different penalty weight for each training path from a uniform distribution over the interval [λ^*l*^, λ^*u*^]. The sampled penalty weight is provided as an additional feature. Again, our experiments show that the sampling procedure improves model performance but fails to evoke conditional behavior. Note of course that it might well be possible to learn goal-conditioned behavior in this setting, e.g., by improving the neural network architecture or hyperparameters. For model evaluation, the target return and penalty weights are not sampled but set to the values μ^eval^ and λ^eval^ across all experiments.

##### 3.2.3.2. Gradient flow

The problem of vanishing and exploding gradients is dealt with by using *gradient clipping* (Pascanu et al., 2013) on all gradients and residual connections (He et al., 2016) in the fully connected layers of the decision network. Gradient clipping helps mitigate the spikes in the loss function that occur due to constraint violations. To further help with the gradient flow in the model, detaching the features from the computational graph before passing them through the neural network leads to a slight improvement in learning. Correspondingly, the forward pass is adjusted to


(48) $$\begin{array}{l} a_{t}=g^{\theta_{t}}(sg\left[X_{t}\right]), \end{array}$$

where *sg* denotes the stopgradient operator.^18^

##### 3.2.3.3. Weight sharing

We also find the optimization to work better when weights are shared between the neural networks, i.e., when setting $g^{\theta_{t}}\equiv g^{\theta }$. This reduces the number of parameters in the model significantly by a factor of *H*−1. It means that each parameter contributes to *H*−1 decisions as opposed to a single decision, which apparently leads to more robust gradients. To provide the model with a sense of time, we provide it with the additional feature


(49) $$\begin{array}{l} X_{t}^{\text{time}}=\frac{t}{T}. \end{array}$$

Leaving out the time feature does not solve the undesired time dependence of decisions that is introduced by assuming a finite time horizon *T* as discussed in Section 2.5. The model can estimate the current time *via* other features but learns quicker if the time feature is provided explicitly.

The decision network *g*^θ^ is trained using the *RAdam optimizer* (Liu et al., 2021). The most important hyperparameters are listed in Table 2. During model development, we trained most models on 40, 000 scenarios for < 100 epochs using *early stopping*. The models used to generate the results reported in Section 4 were either fully trained or at least fine-tuned on constantly resimulated paths. More precisely, we simulated a completely new set of scenarios for each epoch. This is computationally more expensive than training on the same scenarios in each epoch, but it leads to better learning processes.

#### 3.2.4. Implementation

We structured the formulation of the ALM problem in Section 2 into decision-independent and decision-dependent computations since this represents the computational structure of our implementation. All decision-independent computations are made outside of the training loop. While this implementation strategy avoids having to recompute the transition of many model variables in each epoch, it makes the problem memory bound as all the intermediate computations have to be stored and loaded during each epoch. To benefit from GPU acceleration, one needs either a GPU with a lot of memory or needs to size down the batch size, which decreases model throughput and performance. Considering this, it might be faster to recompute even the decision-independent variables during training when computations are done on GPUs (similar to the motivation behind *activation checkpointing*). In addition, computations are slowed by the recurrent structure of the problem, i.e., the loop in Algorithm 1. Decisions in different periods cannot be parallelized as they depend sequentially on each other.

Our implementation is done in *Python*. The decision-dependent transitions and the forward and backward passes through the neural networks (see Figure 2) are implemented in *PyTorch*. For optimization and training code, we use *PyTorch Lightning*.^19^ Using this code, training a full model from scratch on a (weak) CPU takes roughly 8*h*–12*h*, but there is a lot of optimization potential on both the hardware and software sides.

### 3.3. Evaluation

Evaluating our ALM framework is delicate as one has to distinguish between evaluating the problem formulation and the actual performance of strategies conditional on the considered problem. The modeling decisions presented in Section 2 as well as the loss function decisions presented in this section were made in an iterative process: we specified particular premises, learnt and analyzed strategies, and determined whether the results aligned with the many requirements of the bank. In the earlier stages, we often found the learnt strategies to be degenerate in some sense, either exploiting loopholes in the problem formulation or being bound by the strictness of the modeling assumptions. For instance, the bank's legacy financing portfolio includes many short-term maturities that come from opportunities seized during the recent period of negative interest rates. In the initial problem formulation, the shortest financing maturity available was a 3-*y* bond. Once short-term financing had matured, the structure of the balance sheet had to change significantly. Simply rolling these positions over was not available in the action space. This was solved by removing those legacy positions from the model that the bank was seeking to resolve anyway and by extending the available financing maturities to also include 3*m*, 1*y*, and 2*y*. Even after many iterations, there is still some room for improvement for the ALM model. Section 4 compares the influence of different modeling choices in terms of the model horizon *T* and the inclusion of swaps. The most important shortcomings are highlighted in Section 5.

To evaluate strategies within a defined problem setting, we use a collection of metrics that are calculated on *n* validation paths indexed by *i*. To start, we report the loss and loss components that are associated with a given model, namely


(50a) $$\begin{array}{l} \bar{\ell }:=\frac{1}{n}\overset{n}{\underset{i=1}{\sum }}\ell \left(E_{T}^{i},p^{i}|E_{0};\mu ,\lambda \right), \end{array}$$

(50b) $$\begin{array}{l} {\bar{\ell }}^{\text{t}}:=\frac{1}{n}\overset{n}{\underset{i=1}{\sum }}\ell^{\text{t}}\left(E_{T}^{i}|E_{0};\mu \right), \end{array}$$

(50c) $$\begin{array}{l} {\bar{\ell }}^{\text{p}}:=\frac{1}{n}\overset{n}{\underset{i=1}{\sum }}\ell^{\text{p}}\left(p^{i}\right), \end{array}$$

(50d) $$\begin{array}{l} {\text{VaR}}_{\alpha }(\ell^{\text{p}}):\;={\hat{F}}_{\ell^{\text{p}}}^{-1}(\alpha )-{\bar{\ell }}^{\text{p}}, \end{array}$$

(50e) $$\begin{array}{l} {\text{ES}}_{\alpha }\left(\ell^{\text{p}}\right):=\frac{1}{\overset{n}{\underset{i=1}{\sum }}1_{\left\{\ell^{\text{p}}\left(p^{i}\right)\geq {\text{VaR}}_{\alpha }\left(\ell^{\text{p}}\right)\right\}}}\left(\overset{n}{\underset{i=1}{\sum }}\ell^{\text{p}}\left(p^{i}\right)1_{\left\{\ell^{\text{p}}\left(p^{i}\right)\geq {\text{VaR}}_{\alpha }\left(\ell^{\text{p}}\right)\right\}}\right)\\ \;-{\bar{\ell }}^{\text{p}}, \end{array}$$

(50f) $$\begin{array}{l} {\bar{\ell }}^{\text{u}}:=\frac{1}{n}\overset{n}{\underset{i=1}{\sum }}\ell^{\text{u}}\left(E_{T}^{i}|E_{0};\gamma \right), \end{array}$$

where ${\hat{F}}_{\ell^{\text{p}}}$ denotes the empirical distribution function of ℓ^p^(*p*^*i*^) on the validation data set. As we use the target loss (Equation 50b) for the calculation of the total loss (Equation 50a), we also report the mean utility loss component (Equation 50f). Next to the loss metrics, we report metrics that directly characterize the distribution of the *equity ratio* ER: = *E*_*T*_/*E*_0_ on the set validation scenarios. This distribution is denoted by the empirical cumulative distribution function ${\hat{F}}_{\text{ER}}$. We report


(51a) $$\begin{array}{l} \bar{\text{ER}}:=\frac{1}{n}\overset{n}{\underset{i=1}{\sum }}{\text{ER}}^{i}, \end{array}$$

(51b) $$\begin{array}{l} m_{k}:=\frac{1}{n}\overset{n}{\underset{i=1}{\sum }}{\left({\text{ER}}^{i}-\bar{\text{ER}}\right)}^{k},\;k\in \left\{2,3,4\right\}, \end{array}$$

(51c) $$\begin{array}{l} \sigma_{\text{ER}}:=\sqrt{m_{2}}, \end{array}$$

(51d) $$\begin{array}{l} \text{skew}\left(\text{ER}\right):=\frac{m_{3}}{{m_{2}}^{3/2}}, \end{array}$$

(51e) $$\begin{array}{l} \text{kurt}\left(\text{ER}\right):=\frac{m_{4}}{{m_{2}}^{2}}-3, \end{array}$$

(51f) $$\begin{array}{l} {\text{VaR}}_{1-\alpha }(\text{ER}):\;={\hat{F}}_{\text{ER}}^{-1}(1-\alpha )-\bar{\text{ER}}, \end{array}$$

(51g) $$\begin{array}{l} {\text{ES}}_{1-\alpha }\left(\text{ER}\right):=\frac{1}{\overset{n}{\underset{i=1}{\sum }}1_{\left\{{\text{ER}}^{i}\leq {\text{VaR}}_{1-\alpha }\left(\text{ER}\right)\right\}}}\left(\overset{n}{\underset{i=1}{\sum }}{\text{ER}}^{i}1_{\left\{{\text{ER}}^{i}\leq {\text{VaR}}_{1-\alpha }\left(\text{ER}\right)\right\}}\right)\\ \;-\bar{\text{ER}}. \end{array}$$

note the difference in the definition of ${\text{ES}}_{\alpha }\left(\ell^{\text{p}}\right)$ and ES_1−α_(ER): the first metric is defined on a loss distribution and the latter is defined on a P&L distribution. Consequently, both ${\text{ES}}_{0.95}\left(\ell^{\text{p}}\right)$ and ES_0.05_(ER) have the interpretation as how far the mean over the 'worst' 5% of the respective values deviate from the original mean. To compare models across different time horizons *T*, we define the annualized metrics


(52a) $$\begin{array}{ll} \bar{\mu } & :=\frac{1}{n}\overset{n}{\underset{i=1}{\sum }}\left({\left({\text{ER}}^{i}\right)}^{\frac{1}{T}}-1\right), \end{array}$$

(52b) $$\begin{array}{ll} \bar{\delta } & :=\frac{1}{n\left(T-1\right)}\overset{n}{\underset{i=1}{\sum }}\underset{t\in T\backslash \left\{T\right\}\cap \mathbb{N}}{\sum }\frac{\delta_{t}^{i}}{E_{0}}. \end{array}$$

$\bar{\mu }$ denotes the geometric mean return on equity, excluding dividend yield, averaged over all scenarios. $\bar{\delta }$ approximates the annual dividend yield. It is a simplified metric that is meant to give a rough idea of how much dividend yield a strategy provides. Summing the dividends over time is a significant simplification and neglects the time value of money. Furthermore, note that all dividends are standardized by the initial value of equity and not by the equity value of the preceding year.

In the optimization, the interest rate risk is controlled *via* a single number (Equation 25). The IRS captures the risk associated with parallel shifts of the yield curve but does not indicate how the bank's equity would be affected if the shape of the yield curve changed. In ALM, it is therefore common to consider the interest rate sensitivity of the bank's equity separately for each maturity τ∈T: one considers an increase of 100 bps in the yield curve at a single maturity τ and calculates the consolidated impact on equity. The resulting *sensitivity gaps* (also known as *key rate durations*) are aggregated into yearly tranches. By definition, the sensitivity profile reflects cash flows from loans, investments, deposits, and borrowings. Inspecting the sensitivity gaps for different scenarios is extremely insightful since the optimized balance sheet structure and the interest rate exposure is revealed. The model can control the sensitivity gaps *via* the investment and financing portfolios.

## 4. Experiments and results

There are many interesting questions to pursue with the precise model of the ALM problem and the Deep ALM method for solving the problem. In this article, we restrict ourselves to three aspects. First, we demonstrate that the Deep ALM method works and outperforms the given benchmarks. Second, we analyze the learnt strategies on a selection of validation scenarios that differ in the evolution of the yield curve, including *steepening* and *inversion*. Third, we analyze how the extension to swaps affects the learnt strategies and ultimately the bank's P&L. Throughout this analysis, we compare two different settings of the ALM problem that differ in terms of the modeled time horizon *T* = 5*y* and *T* = 15*y*. 15*y*|5*y* refers to as modeling and optimizing with respect to 15*y* and evaluating the strategy already at the 5*y* horizon.

### 4.1. Main results

The following section focuses on the general performance of the Deep ALM strategy and the benchmark strategies. All models are trained separately in the two different settings of *T* = 5*y* and *T* = 15*y*. The performance of the strategies is evaluated on a validation set of 1600 yield curve scenarios using the metrics defined in Section 3.3. The results are reported in Table 3. The main conclusion that can be drawn from these results is that models with a larger number of trainable parameters perform better than those with fewer parameters. In particular, Deep ALM outperforms all benchmarks significantly. This can be observed unanimously across all metrics: the main model has lower loss statistics and a more favorable equity distribution than the benchmarks. This indicates that there is a decent alignment between the loss function and the underlying preferences.

The loss statistics indicate that in the 5*y* setting, even the simple benchmarks seem to be able to adhere to constraints in most scenarios as the incurred penalty loss is small. The main model only has a very small penalty loss and seems to stay within the constraints even better. In the 15*y* setting, the benchmark strategies incur large penalty losses, but the main model achieves a small penalty loss. This gives the indication that constraints are more difficult to comply with in the 15*y* setting. Consequently, they have a bigger impact on the loss function leading to different strategies when compared to the 5*y* horizon.

**Figure 7:**
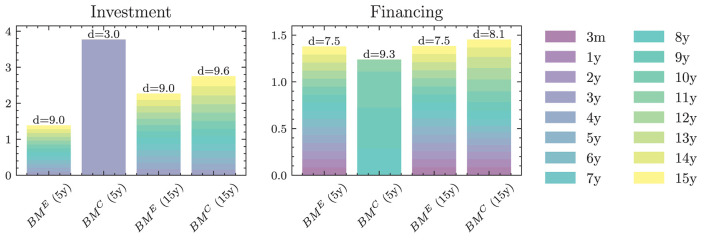
Constant benchmark strategies—The vertical axis denotes the scales of total investment and financing relative to the maturing positions, i.e., $∥a_{t}^{B}{∥}_{1}/\pi^{\left(1\right)}(B_{t})$ and $∥a_{t}^{K}{∥}_{1}/\pi^{\left(1\right)}(K)$. The variable *d* above the columns specifies the duration of the portfolios.

The distribution of the final equity ratio under the Deep ALM strategy is more favorable compared to the distribution obtained under the best benchmark *BM*^*D*^: following *MM*, one obtains a distribution of ER = *E*_*T*_/*E*_0_ that has a higher mean with lower risk; both value at risk and expected shortfall are smaller. The distribution also has a more positive skew, but a slightly larger standard deviation when compared to the benchmark. Looking at the right tail of the equity ratio distribution under the *MM* in Figure 7, one can observe that there are a number of validation scenarios in which the model achieves a much better equity ratio than in the mean. The distribution of ER does not seem to be fat-tailed on the left. This asymmetry is due to the loss function that penalizes downward deviations from the targeted equity ratio but does not penalize upward deviations.

#### 4.1.1. Benchmarks

**Figure 8:**
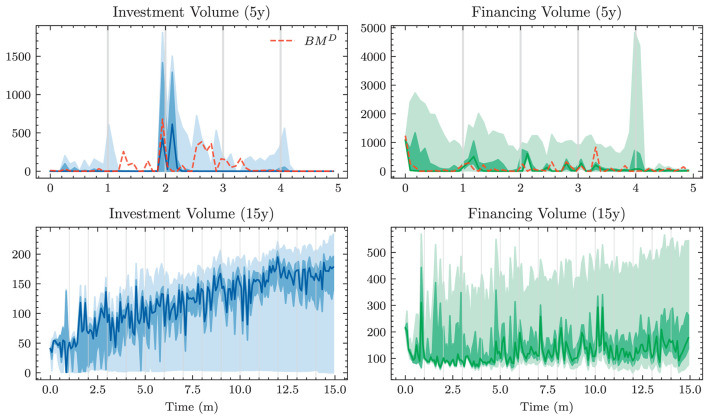
Investment and financing volume—Investment volume refers to $∥a_{t}^{B}{∥}_{1}$ and financing volume refers to $∥a_{t}^{K}{∥}_{1}$. The solid line indicates the median level of $∥a_{t}^{B}{∥}_{1}$ or $∥a_{t}^{K}{∥}_{1}$ across all 1 600 validation scenarios. The darker shaded area is enclosed by the 25% quantile and the 75% quantile. The lighter shaded area is enclosed by the 5% quantile and the 95% quantile. The gray vertical lines indicate the times at which the annual step occurs, i.e., dividends are paid out and the minimum return constraint is checked. The same layout is used for all plots in this section.

Visualizing the learnt strategies in Figure 8 leads to a couple of interesting observations. First, we see that all benchmarks invest significantly more than the maturing amount. The benchmarks also borrow more than the maturing amount, but borrowings are scaled up less than investments. In absolute terms, financing is still able to cover a lot of the investment activities as the initial financing portfolio is much larger than the initial investment portfolio. In the case of strategy *BM*^*E*^ over 5*y*, investments are made at such a large scale that the cash position is reduced significantly over time. In the case of the other three depicted strategies, the cash position is slightly increasing over the model horizon. This points to the main observation of the benchmark experiments: the learnt strategies in the 5*y* and 15*y* settings differ significantly. *BM*^*C*^ over 5*y* suggests investing at shorter maturities than financing, while *BM*^*C*^ over 15*y* suggests investing at slightly higher maturities than financing. For the purpose of providing a good default ALM strategy, this one trained to optimize long-term goals seem to be more relevant than the 5*y* strategy. The clear dependence of the benchmark strategies on the time horizon *T* motivates analyzing how the Deep ALM strategies depend on the very choice.

#### 4.1.2. Deep ALM

**Figure 9:**
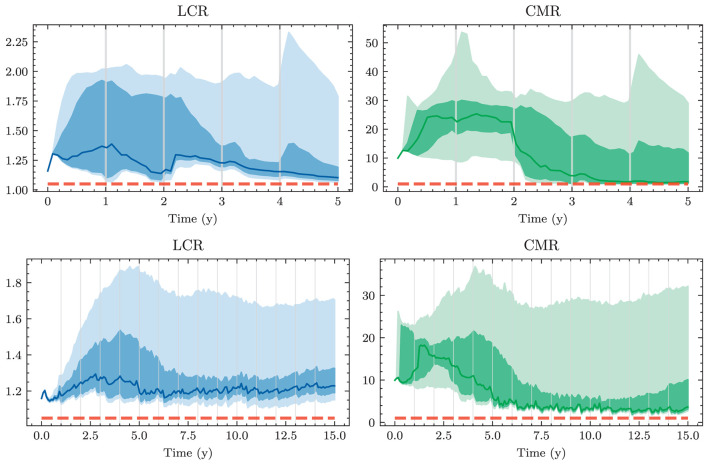
LCR and CMR.

Visualizing and interpreting the Deep ALM strategies is difficult because decisions now differ based on the scenario and model time. We start by considering only the volume of investments and borrowings, shown in Figure 9. One can observe that for both time horizons, the learnt strategies are generally scenario-dependent. Still, the quartile lines of the volume plot show that the strategies chosen in different scenarios are, at least in terms of volume, often quite similar. This comes at no surprise given that many scenarios are quite similar. By deviating from the median strategy in the other scenarios, the model generates the outperformance of the benchmark *BM*^*D*^ observed in Table 3. Interestingly, the median strategy does not correspond exactly to the benchmark strategy *BM*^*D*^ from which the model learns to deviate.

Second, we see that the strategies optimized on the different time horizons are significantly different. In the case of the 5*y* horizon, there are many periods in most scenarios where no investments are made at all. At the 2*y* mark, investment activities spike. In Section 4.3, we will see that these investments are always made at the 3*y* maturity. Thus, 3*y* before the model horizon ends, the model makes large investments into 3*y* bonds, again pointing toward the role of the model horizon *T*. Concerning financing activities in the 5*y* setting, one can observe that both the benchmark *BM*^*D*^ and the main model raise funds of roughly *m*CHF 1, 000 in the first model period. This decision is taken in each scenario as all scenarios have the same initial state. Raising funds in the first period but not making any investments builds up a cash position and increases the liquidity ratios LCR and CMR. Withholding cash from investment is usually undesirable when interest rates are positive as the bank does not earn any interest on the cash position. This decision seems to be motivated by the expectation that interest rates increase over the first few model periods. If the yield curve shifts upwards, investment and financing portfolios decrease in value. In that case, taking on additional financing before rates increase and keeping these funds in cash leads to an increase in equity. This highlights the impact of the yield curve scenarios on the learnt strategies. The fact that interest rates are increasing in most scenarios (see again Figure 4) induces the model's expectation of increasing rates and the scenario-independent decision of raising large funds in the first period.

**Figure 10:**
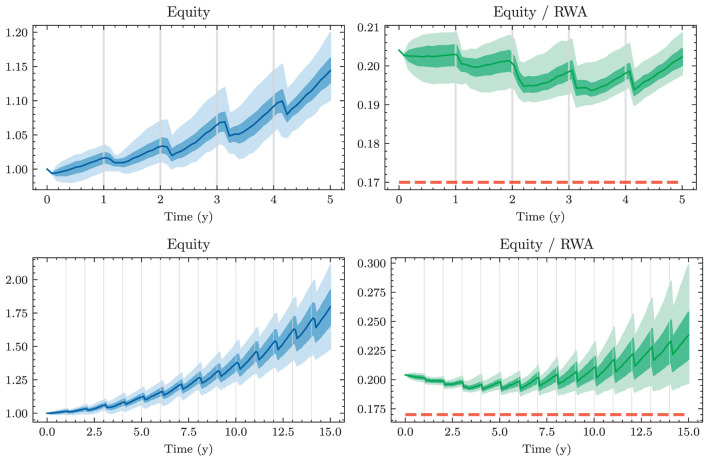
Equity/RWA—Note that the dividend payouts lead to downward jumps in the bank's equity. In the figures, this impact seems to be delayed by one period. This is because the equity value is recorded at the end of the restructure step; but the annual step is performed after the balance sheet restructuring.

**Figure 11:**
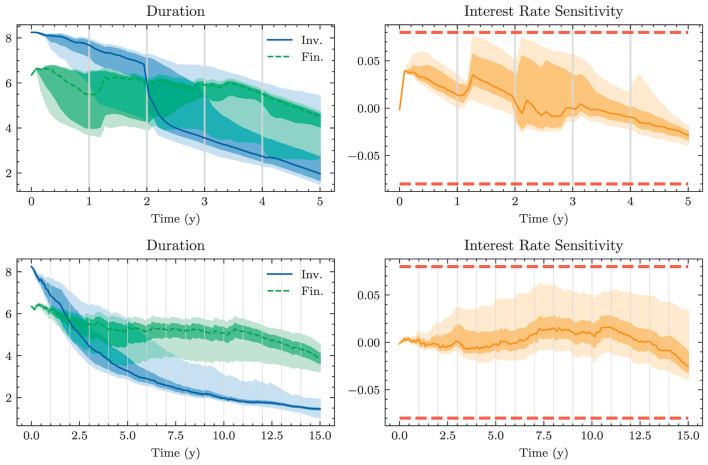
Interest rate sensitivity—Whereas the durations in this chart only account for parts of the balance sheet (i.e., the duration structure of the balance sheet will not necessarily be inverted by the end), the interest rate sensitivity is measured holistically.

In the 15*y* setting, investment and financing decisions are more equally distributed across time. Each month the model invests and borrows at a similar scale as in the previous month. Spikes in investment and financing volumes are much lower than in the 15*y* setting. This continuous investment and financing pattern looks quite similar to ALM strategies pursued in practice. In the first few model periods, one can observe a similar strategy as pursued in the 5*y* setting: borrowings often exceed the amount needed to finance investments and new loans, leading to the buildup of a larger cash position; see also the median CMR in Figure 10. Again, this likely corresponds to the expectation of increasing interest rates during the first periods. As time evolves, the median investment volume increases continually, which is sensible considering that the other balance sheet items grow as well. The median financing volume also starts increasing after the fifth year, but it increases less in relative terms. This is in line with the observation that the model restructures the bank toward less leverage in the long term; see Figure 11.

### 4.2. Constraints

Table 4 reports detailed statistics on the constraints. In both the 5*y* and 15*y* settings, the main model is able to adhere to the five regulatory constraints in almost all cases. The NSFR constraint is never violated and does not seem to affect the model decisions. The model typically borrows more funds than necessary to roll over the legacy portfolios, which increases the amount of stable funding. While increases in investments lead to an increased required amount of stable funding, the model never increases investments to a level that would destabilize long-term liquidity. In fact, a brief inspection showed that the NSFR never dropped below 130% in at least 90% of the scenarios. Qualitatively, the movements are similar to those of the LCR.

**Table 4:** Constraint statistics.

**Constraint**	**CMR**	**LCR**	**NSFR**	**E/RWA**	**IRS**	**EYR**
*MM* **(5y)**						
Median at *T*−1	1.76	1.10	1.33	0.20	−0.03	21.17
% of scenarios with violations	0.12	0.00	0.00	0.00	0.06	20.06
Mean number of violations per violation scenario	1.00	-	-	-	1.00	1.08
Mean violating value	0.94	-	-	-	0.09	−2.99
Value at largest violation	0.92	-	-	-	0.09	−23.80
*MM* **(15y)**						
Median at *T*−1	3.66	1.23	1.34	0.24	−0.03	63.43
% of scenarios with violations	0.12	0.00	0.00	0.12	0.12	45.94
Mean number of violations per violation scenario	1.00	-	-	2.50	4.00	1.35
Mean violating value	0.91	-	-	0.17	0.09	−5.54
Value at largest violation	0.85	-	-	0.16	0.09	−53.68

LCR and CMR seem to have a much bigger impact on constraining liquidity. In the case of the 5*y* horizon, both ratios increase in the first period due to the increased borrowing that occurs in each scenario. Over the course of the next periods, the model further increases both ratios in most scenarios before either of them decreases over the remainder of the model period. After 5 years, both median ratios lie only slightly above their respective minimum boundaries. Still, the model avoids violations of the LCR and CMR constraints in all and more than 99% of scenarios, respectively. Interestingly, the behavior is different when optimizing over the long-term horizon. The LCR is held at a median level that is above its initial value and not decreased as the model period approaches the end. The CMR decreases in most scenarios over the model period but is sustained at a higher level than in the 5y optimization. On the 5*y* horizon, the model can reduce its cash position to the bare minimum and avoid constraint violations at the same time. On the 15*y* horizon, this becomes unsustainable, and the model instead decides to keep a larger cash position to remain compliant. This interpretation again indicates that the 15*y* setting seems to have a better alignment with the true preferences of the bank. The observation that the cash position is reduced in most of the scenarios is meaningful considering that the large initial cash position is a remainder from the recent period of negative interest rates. Unwinding this cash position is beneficial as interest rates are positive in most scenarios.

The model adheres to the leverage constraint on the ratio between equity and RWA in all and more than 99% of the scenarios in the 5*y* and 15*y* setting, respectively. In both cases, this ratio first slightly decreases as RWA grow quicker than equity due to increased investments. Toward the end of the first 5 years, increases in equity translate into an increase in the ratio, away from the lower bound of 17%.

The model is also able to comply with the interest rate sensitivity constraint, only violating it in one and two validation scenarios in the 5*y* and 15*y* settings, respectively. It seems that the restrictions imposed by other constraints, especially the EYR constraint, imply that interest rate risk must be hedged to such a degree that the model almost never attempts to push the sensitivity constraint to its boundaries. In the 5*y* setting, the extensive initial borrowing corresponds to a positive IRS strategy: because the model expects interest rates to rise, it wants to have positive exposure to such movements. As the model time progresses, the IRS decreases and becomes negative during the fifth and final year. The IRS strategy followed in the 15*y* setting is more cautious as the IRS is kept around 0% for much of the model period, before also being reduced to a negative level during the last 3 years. The fact that we can observe the model pursuing a negative IRS in only the later parts of both model periods points again toward the impact of the model horizon.

**Figure 12:**
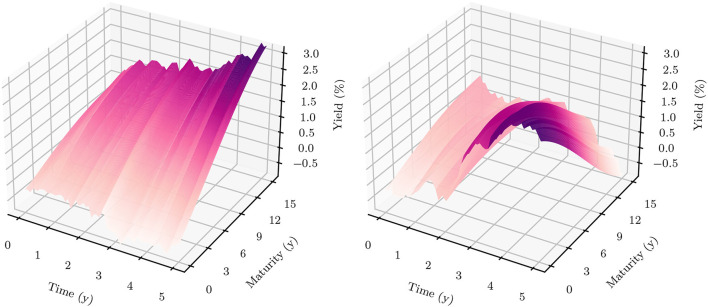
Yield curve scenarios (5*y*)—Scenarios *steep* on the left-hand side and *inversion* on the right-hand side.

The model can control the IRS *via* the size and duration of the investment and financing portfolios. In both settings, the model decreases the duration of the investment portfolio over time. In Figure 12, we see that this is achieved by investing mainly in short-term bonds and letting longer portfolio positions mature. Notice that the duration of the investment portfolio develops similarly in both horizon settings when viewed on the relative time scale *t*/*T*. This again points toward an undesired impact of the time horizon *T*: if we learnt “the” best ALM strategy, both would follow the same strategy on the absolute time scale. The duration of the financing portfolio decreases slightly over the majority of the model period, before decreasing more sharply at the end of the model period. Again, one can observe that the financing duration evolves similarly in both settings when viewed on the relative time scale. This effect is *not overfitting* in the classical sense. In the training process, a strategy is learnt that optimizes the objective of expected utility maximization at the selected end point. In this process, the strategy can be generalized from the training data to the validation data (which would not be the case with classical overfitting). However, this overengineering with respect to the terminal model point is not practical for the bank as a going concern. It is rather due to the limitation of the chosen DSC approach, which requires a cut-off date for optimization. This is a misalignment between the 'short-term' optimization problem and real-world ALM. The strategy of choosing a reference point for optimization far in the future and then limiting the analysis and conclusions to a shorter time window seems to be a viable solution to address this issue.

The constraint that is violated most often is the constraint on the minimum annual return. This is unsurprising considering that adhering to this constraint is considered less important than adhering to the regulatory constraints. Consequently, the relative weight σ_*i*_ placed on this constraint is lower than that placed on the other constraints. Beyond its lower prioritization, the constraint on the EYR seems to be a particularly difficult constraint to adhere to. Additional experiments showed that constraints that are easy to comply with will rarely be violated even if the assigned penalty weight is relatively small. This is not the case for the EYR constraint, which essentially requires that the bank never encounters an adverse year, even if the interest rate environment changes to great effect. The number of violations in the 5*y* and 15*y* settings is roughly equal when adjusted for the length of the period. This is remarkable considering that the variety of yield curve shapes and levels attained over the 15*y* horizon is larger than that on the 5*y* horizon. The greater yield curve diversity makes adherence to the EYR constraint more difficult. This is evidenced by the fact that the benchmark strategies are often able to comply with this constraint over 5*y* but not over 15*y*; cf. the large penalty losses in Table 3. The fact, that the *MM* (15*y*) strategy manages to comply with the EYR constraint most of the time, indicates that the 15*y* strategy is generally more cautious. This explains the large differences between the 5*y* and 15*y* strategies: while a strategy that is close to the constraint limits works on the 5*y* horizon, it becomes unsustainable in the long run.

### 4.3. Scenario analysis

#### 4.3.1. 5*y* horizon

**Figure 13:**
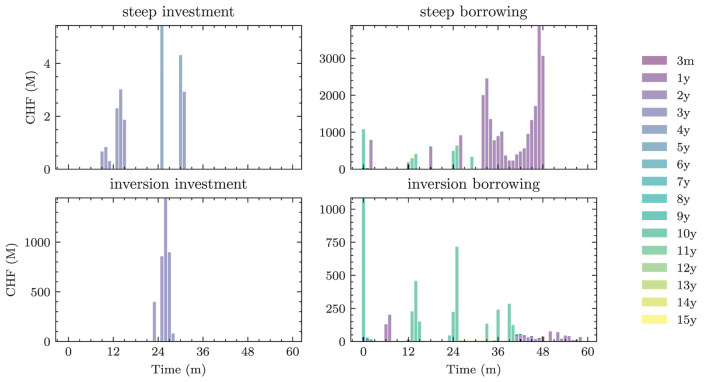
Decisions (5*y*).

We now analyze the strategies of the main model *MM* on two particular yield curve scenarios depicted in Figure 13. Both scenarios are part of the validation set of yield curve scenarios simulated using the HJM-PCA model. They were chosen as illustration examples because of the prototypical yield curve movements. In the first scenario, referred to as *steep*, the yield curve steepens and in the second scenario, referred to as *inversion*, the yield curve inverts during the second half of the model period.

**Figure 14:**
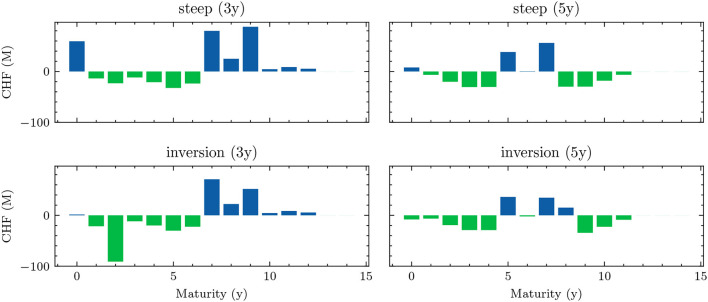
Sensitivity gaps (5*y*).

Focusing on a single scenario at a time gives us the chance to visualize the exact model decisions taken; see Figure 14. In the steepening scenario, the model only makes negligible investments over the entire period. During the first 3 years of this scenario, the yield curve barely changes. Even after 2 years, where we typically find large investments in the 5*y* setting, the model only invests tiny amounts. On the liability side, the model borrows a reasonable amount at short (3*m*) and long (10*y*) maturities during the first half of the model period. In the second half of the model period, it is common among many scenarios that the model raises short-term financing. In this scenario, the borrowed amounts are very large.

In the inversion scenario, one can observe the typical investment strategy alluded to earlier: 3 years before the end of the model horizon, the model invests heavily into 3*y* bonds. At that time, the yield curve has not yet inverted and lies slightly below the initial yield curve. After the subsequent yield curve inversion, no more investments are made. Funds are mainly borrowed at the 10*y* maturity during the first 3.5 years. In the last 2 years, we can observe the typical pattern of short-term financing. When compared to the steepening scenario, the volume of short-term financing toward the end is much lower, which is reasonable considering that short-term financing is expensive under the inverted yield curve.

**Figure 15:**
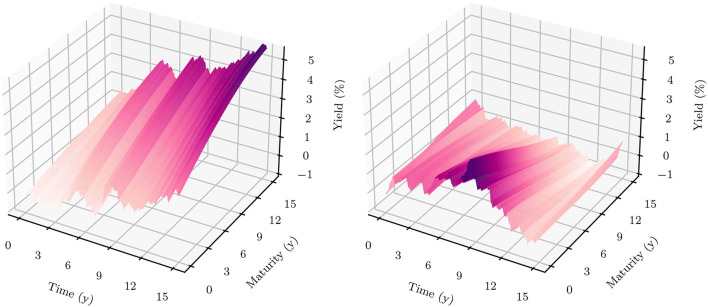
Yield curve scenarios (15*y*)—Scenarios *incr* on the left-hand side and *inv_and_back* on the right-hand side.

In both scenarios, the model follows its default strategy during the first 2 years: withhold investments and refinance with mostly 10*y* bonds whenever the IRS turns negative. As pointed out before, this behavior is likely due to the expectation of increasing interest rates. Considering that in both scenarios, yield curves stay relatively constant during that period, observing similar behavior should be expected. It seems less clear why the model then decides to follow its usual strategy of making large investments in 3*y* bonds in the inversion scenario, but not in the steepening scenario. A possible explanation might be the difference in the 3*y* yield. In the inversion scenario, the yield curve takes a steep upward shift starting around the 24^*th*^ month, such that investments made afterwards earn higher interest rates. In the steepening scenario, the yield curve after 2 years is very similar to the initial yield curve, which carries a 3-*y* yield that is only slightly above zero. Thus, the benefit of making a 3-*y* investment over holding cash is marginal. This is important because the model only seems to consider investing in the shortest maturity. While this restriction to short-term investments may be related to the adverse effect of the 5*y* horizon, it can also be observed in the 15*y* setting mentioned later. This indicates that the focus on short-term investments is motivated by other factors such as compliance with the EYR constraint as discussed in the next section. In the steepening scenario, the IRS sensitivity profile is, without making any investments, well set up for the steepening in the yield curve. Figure 15 shows that especially due to the 10*y* borrowings made in the previous periods, the interest rate sensitivity is positive for the maturities 7*y*–10*y*. At most other maturities, sensitivities are much smaller in absolute terms. This means that a steepening in the yield curve leads to an increase in the bank's equity.

#### 4.3.2. 15*y* horizon

**Figure 16:**
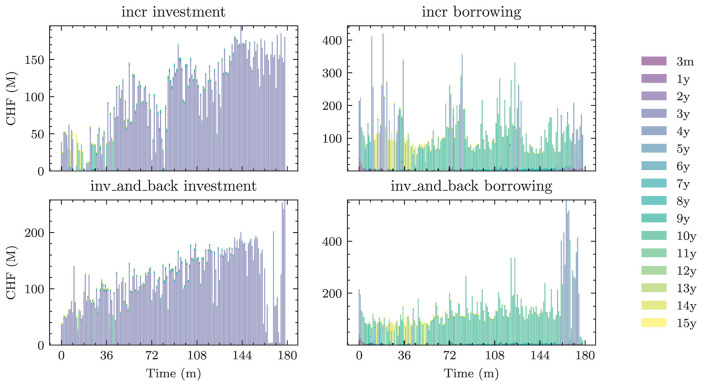
Decisions (15*y*).

On the long time horizon, selecting a “reasonable” or insightful yield curve scenario for the analysis becomes more difficult and interpretations should be drawn carefully. Again, two scenarios are selected from the validation set which are simulated using the HJM-PCA model; see Figure 16. In the first scenario *incr*, the yield curve generally shifts upwards and steepens over the entire model period. Roughly in the sixth and the eighth year, the yield curve shifts downward two times for a while before continuing its upward trend. In the second scenario *inv_and_back*, the yield curve changes its shape multiple times throughout the 15 years. The yield curve flattens over the first periods, then inverts and shifts upwards, before yields decrease and flatten again. At the end of the 15 years, the yield curve steepens.

In the first scenario, the yield curve stays roughly at its initial level for the first 3 years. During that time, the model slowly builds up a slightly positive interest rate sensitivity, likely due to the model anticipating increasing yields. This is achieved *via* a low investment volume and an increased financing volume. Both investments and borrowings are made using a mix of short- and long-term maturities. For the remainder of the period during which the yield curve generally increases and steepens, investments are made at the shortest investment maturity of 3*y*, and financing is mostly done at the 10*y* maturity. During the two periods where yields are slightly lower, investments decrease, and total borrowings increase, with some of these borrowings made at shorter maturities. This strategy increases the IRS leading to the interpretation that the model expects the yield curve to increase again. Toward the end of the model period, we observe that long-term financing is replaced with short-term financing. This observation can be made in both scenarios, again pointing toward the adverse effect of the time horizon *T*. Decisions in the second scenario are generally quite similar to the first scenario. Investments are mostly made at short-term maturities. Financing is mainly done at long-term maturities in the first periods, at the 10*y* maturity between the 5th up until the 13th year, and at short-term maturities in the final periods. Because the yield curve is inverted for much of the period, these decisions build up a larger IRS than in the first scenario.

**Figure 17:**
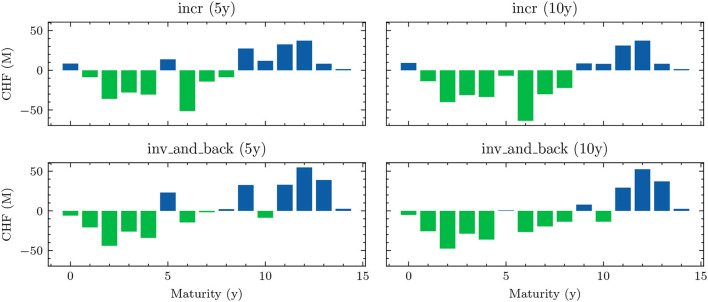
Sensitivity gaps (15*y*).

**Figure 18:**
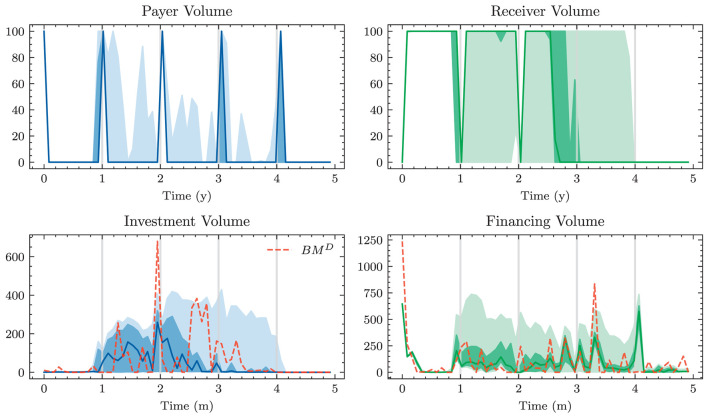
Swap volumes—Note that the bond volume refers to the value of bonds purchased and issued in *m*CHF. The swap volume refers to the notional amount in *m*CHF of swaps entered into this period. The swap value is, aside form spreads, zero at issuance.

The focus on 10*y* borrowing in both scenarios of Figure 17 is likely due to the fact that approximately 40% of new mortgages are assumed to have a 10*y* maturity. Apparently, the model does not want to finance these loans with short-term deposits and build up a negative sensitivity with respect to the 10*y* yield. Instead, it raises funds to finance a majority of these loans at the same maturity. Figure 18 displays that the 10*y* absolute sensitivity is comparatively small. Because customers pay a larger spread on loans than the bank pays on their financing, the model detects a risk-free profit with this strategy limited by the volume of the loans. This behavior of directly financing new loans with additional financing can only be consistently observed at the 10*y* maturity. New loans that are granted at other maturities (mostly at maturities < 10*y*) are likely financed to a large extent by deposits. Since these do not match perfectly in maturities, interest rate risk arises. Hence, the model specifically offsets interest rate risk at some maturities but keeps exposure at others.

Figure 18 shows the gap structure in the two 15*y* scenarios at two points in model time, namely just after 5 and 10 years. The gap profile observed in the two different scenarios is quite similar when compared at the same point in time. The 10*y* sensitivity is of small absolute size as a majority of the sensitivity arising from the 10*y* mortgages is hedged *via* 10*y* financing. The tranches up to a maturity of 10 years always have a negative sensitivity, except for the first and the sixth tranche which either have a positive or small negative sensitivity. Tranches with a maturity of 10 years and above mostly have a positive sensitivity.

The sensitivity profile chosen by the model may be interpreted as follows. First, the overall sensitivity that the model chooses is limited: all sensitivity gaps are relatively small in absolute terms and the netted sensitivity gaps are often close to zero, which corresponds to an almost vanishing IRS. Within the tranches up to maturities of approximately 7 to 8 years, the model pursues a strategy of positive maturity transformation to profit from upward sloping yield curves. The negative sensitivities arise from loans and investments at these maturities that are partially financed with short-term debt such as non-maturing deposits. Note that in the inversion scenario, where maturity transformation might lead to losses^20^, the mid-term sensitivities are smaller in absolute terms. The fact that maturity transformation is exploited at the short- and mid-term maturities (instead of long-term investments) might be due to the fact that the initial yield curve and many simulated yield curves are steepest on the short end, making the maturity transformation at short maturities more profitable. At the same time, long-term investments would build up sensitivities that are in absolute terms much larger than the short-term investments. This would thus lead to an overall IRS that is negative and closer to the boundaries than that pursued by the model. This would also make compliance with the minimum annual return constraint more difficult, which penalizes equity volatility. Additional experiments confirmed that when decreasing the weight associated with violations of the minimum annual return, the model chooses more long-term investments, even though a majority remains still short term. This results in a higher volatility of the bank's equity across model time, but a higher mean equity at the end of the period. The impact of the penalty weight illustrates the importance of tuning the loss function parameters to preferences and requirements.

Not only does the model not invest long-term, but also loan volumes are small at maturities over 10 years. Nonetheless, one can observe that in both scenarios the model consistently borrows small volumes at the 15*y* maturity. Because the amount of assets at these maturities is so low, even small financing positions build up positive sensitivity gaps at the long-term maturities. These positive sensitivity gaps offset the negative sensitivities at the short- and mid-term maturities and keep the net IRS low. The low volume needed to create these gaps means that the hedge achieved with these positions is relatively cheap. In addition, 15*y* yields are in many of the simulated scenarios not much larger than 10*y* yields, i.e., the yield curve flattens toward the far end. This makes long-term financing at small volumes attractive.

### 4.4. Long-term optimization and short-term validation

The 15*y* optimization seems to have a better alignment with actual objectives in ALM. But interpreting and selecting strategies solely based on their success after many years is difficult and individual analyses are impractical when facing a large set of yield curve scenarios. This section presents two further approaches that help with model interpretation.

#### 4.4.1. Intermediate analysis

For a practical application, it is important to understand how well the bank is performing in the mid-term, when following a strategy that optimizes for long-term success. To this end, the strategy *MM* (15*y*), which has been trained on the 15*y* horizon, is evaluated on the 5*y* yield curve scenarios. This is achieved by stopping the forward pass through the entire computational graph early, i.e., shortening the loop in Algorithm 1 from *H* = 180 to *H* = 60. Recall that weights are shared in the 15*y* model and the relative time feature *t*/*T* is a model input. When evaluating on *T* = 5*y*, the model time is provided relative to *T* = 15*y*, such that the time feature refers to the same points in time during training and evaluation. The performance of the 15*y* model on the 5*y* horizon is reported in Table 3 in column *MM* (15*y*|5*y*). As expected, the 5-year performance of the 15*y* strategy is worse than that of *MM* (5*y*). This highlights that the practically undesirable strategies pursued by the 5*y* model are not the result of a failed optimization but rather due to a misspecified problem setting. The 5-year performance of the 15*y* model is comparable to the performance of the best benchmark strategy *BM*^*D*^ (5*y*). The 15*y* model is better than the benchmark strategy in complying with constraints. However, this comes at the cost of a higher VaR and ES in the equity ratio distribution after 5 years. This observation is in line with the objective of the 15*y* optimization which penalizes constraint violations in all periods but does not reward the exact equity distribution in any intermediate period. As indicated in Section 2.5, assigning rewards annually based on the equity ratio distribution is an alternative objective which is worth pursuing in the ALM framework. When applying the 15*y* model on the two 5*y* scenarios considered before, decisions are, as they are made from the same model, generally comparable to the decisions observed in the first five years of the 15*y* scenarios. Investment and financing are done consistently each month with slightly increased financing and decreased investment in the first periods. Most investments are made at the 3*y* maturity, financing is mostly done at the 10*y* maturity in the inversion scenario and at a mix of maturities in the steep scenario. The *MM* (15*y*|5*y*) is thereby very different from the *MM* (5*y*) strategy: there are no spikes in investments after 2 years and there are no large short-term borrowing positions toward the end of the 5 years. In equity terms, the performance is similar on a steepening yield curve but worse for an inverting term structure.

#### 4.4.2. Scenario categorization

So far, the performance analysis has been conducted on a high level (full validation set) and on a detailed level (single validation scenarios). The following demonstrates how a medium granularity can offer new insights into model performance. Based on the yield curve movement, we categorize some of the 1600 validation scenarios into five subsets containing each 50 scenarios. In particular, these five categories are considered:

– *Steep:* Scenarios for which the *steepness* of the final yield curve, measured by subtracting the 1*m* yield from the 15*y* yield, is maximal.
– *Up:* Scenarios for which the 1*m* yield lies above 2% and the steepness of the final YC is maximal; the second condition prevents only flat yield curves being considered, which are the most common shape for yield curves equipped with high short-term yields.
– *Down:* Scenarios for which the average yield at time *T* across all maturities is minimal.
– *Inversion:* Scenarios for which the steepness of the final yield curve is minimal.
– *Constant steepness:* Scenarios for which the steepness of the yield curve has the lowest standard deviation across the model periods.

The models are evaluated on these subsets. Table 5 reports the CRRA utility loss and the penalty loss achieved by the different models in the five yield curve categories. Both loss statistics show that there are significant differences in performance between the different yield curve scenarios for any particular model. All models seem to have similar strengths and weaknesses: in terms of utility, they all perform best in the down category and worst in the up category. This might be due to the fact that all models choose strategies with a negative IRS toward the end of the model period; see Figure 12. Hence, if yields decrease right before the end of the period, the bank's equity increases. In contrast, if yields increase right before the end of the model period, equity decreases. When comparing the different strategies with each other, Table 5 highlights again that the *MM* (5*y*) outperforms the *MM* (15*y*|5*y*) with respect to both metrics. Similarly, one can again observe that both main models incur significantly lower penalty losses than the most competitive benchmark strategy. The main models mostly incur penalty losses in the up and inversion scenarios, where large moves in the yield curve likely lead to a violation of the EYR constraint.

**Table 5:** Category statistics—Note that both loss functions are averaged over the 50 scenarios within each category.

**Category**	**Steep**	**Up**	**Down**	**Inversion**	**Constant steepness**
${\bar{\ell }}^{\text{u}}\left(\gamma =10.0\right)$					
*BM*^*D*^ (5*y*)	–0.071	–0.063	–0.086	–0.074	–0.074
*MM* (5*y*)	–0.085	–0.066	–0.096	–0.079	–0.079
*MM* (15*y*|5*y*)	–0.079	–0.048	–0.093	–0.064	–0.075
${\bar{\ell }}^{\text{p}}$					
*BM*^*D*^ (5*y*)	0.050	0.011	0.113	0.022	0.006
*MM* (5*y*)	0.001	0.008	0.005	0.010	0.000
*MM* (15*y*|5*y*)	0.001	0.026	0.003	0.033	0.002

The results of the categorical yield curve analysis on the 5*y* horizon should be interpreted carefully. First, the selection criteria were largely based on the final yield curve. Yield curve paths could be quite different among scenarios from the same category. Second, on such a short horizon, yield curve movements have a large impact on the valuation of cash flows on the bank's balance sheet and a small impact on the cash flows themselves. For instance, legacy loans still have a large impact and pay interest rates that were determined in the past. If interest rates stay at a given level for a longer period, interpretations may change drastically.

### 4.5. Extension to swaps

This section presents results in the 5*y* setting extended with swaps.^21^ Table 3 reports the evaluation metrics obtained by the Deep ALM strategy in the extended model. The swap strategy *MM*^*S*^ outperforms the strategy *MM*, which does not have access to swaps, across all the relevant metrics. Note that the reported mean loss $\bar{\ell }$ and mean target loss ${\bar{\ell }}^{\text{t}}$ should not be compared between the two models because the swap model is trained with a higher target return μ. We find that to achieve the best performance (in terms of the other metrics) in the swap setting, one should be more ambitious and target a higher return.

**Figure 19:**
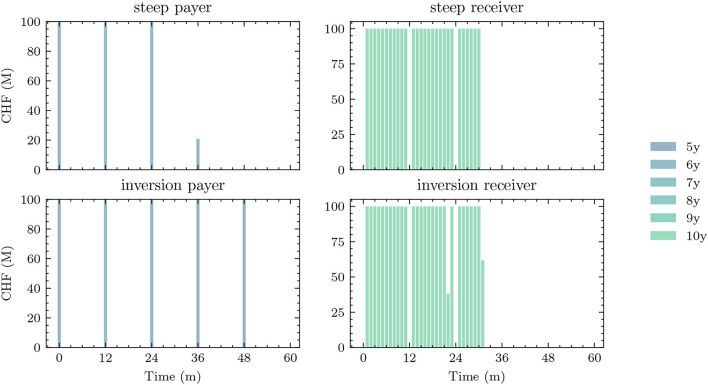
Swap decisions (5*y*).

The swap decisions made by strategy *MM*^*S*^ are the same in most of the scenarios. In the case of receiver swaps, the model decides to enter into as many swaps as allowed by the restrictions on the swap volume. Consequently, the model tries to build up the maximum allowed swap position of *m*CHF 2800 as quickly as possible. Because monthly swap volumes are capped at *m*CHF 100, the model builds the maximum receiver swap position by entering a receiver swap with a notional amount of *m*CHF 100 in each of the first 28*m*. Looking at the volume of entered swaps in Figure 19, one can see that there are some months where the model does not enter receiver swaps but decides to enter payer swaps. Once more, the model maxes out the monthly cap of *m*CHF 100. The months during which the model enters into payer swaps are spaced annually such that the exchange of cash flows occurs immediately before the annual step, when the dividends are issued, and the minimum return constraint is assessed. These payer swaps seem to be used as a hedge against increasing 1*y* interest rates. In the two considered example scenarios in Figure 19, the model enters into receiver swaps at the longest possible maturity of 10*y* and enters into the payer swaps at the shortest available maturity of 5*y*. These maturity decisions can be observed across the majority of scenarios.

The portfolio of receiver swaps is built to pursue positive maturity transformation. Instead of constructing this *carry trade via* investing in bonds (or lending) and borrowing from bonds (or from customers), the model enters 10*y* receiver swaps. On these, the bank receives a fixed rate that depends on the 1*y*–10*y* interest rates at issuance time and has to pay the floating 1*y* rate. Hence, if the initial yield curve has a positive slope and stays constant afterwards, the bank collects a premium associated with the positive slope in the yield curve. The model prefers using receiver swaps to construct the carry trade instead of using bonds because the receiver swap is much cheaper. When borrowing funds at short maturities and investing them at longer maturities the bank has to pay a total spread of 2 × 15 bps = 30 bps, whereas the receiver swap can be entered into at a spread of 2 bps. These more favorable market conditions are also likely the reason why the model pursues the maturity transformation *via* receiver swaps so strongly. On the other side, investment and borrowing volumes are now much lower when compared to the setting without swaps. Interestingly though, the investment and financing strategies that accompany the (almost) scenario-independent swap strategy vary across scenarios. The model uses investments and borrowings for the necessary adjustments that make the constant swap strategy work in every scenario.

Still, it seems unlikely that the optimal swap strategy is almost independent of the yield curve scenario. First, note that the quick accumulation of swap positions should be interpreted with the caveat that the bank is not assumed to hold any legacy swap portfolios, which is not the case in practice. Second, these results only show the low scenario-dependence up to the volume constraints. Additional experiments found that if the volume caps are removed, swap strategies are much more scenario-dependent, but the model builds up impractically large swap portfolios. At the same time, the extreme scenario independence of the swap strategy might be due to a learning issue. In the setting without swaps, one could commonly observe that whenever models did not learn well, they often resorted to constant strategies across all scenarios, a strategy captured *via* the benchmark *BM*^*D*^. Learning that the constant swap strategy leads to good results across all scenarios is much simpler than learning when it might make sense to deviate from this strategy. Nonetheless, the results in the 5*y* should also not be overinterpreted, considering that the 5*y* setting also leads to unintuitive results when no swaps are available. This does not change the interpretation that building up a portfolio of receiver swaps is a sensible strategy for the bank in the current interest rate environment. A similar strategy including the unwinding of a payer swap portfolio (which is a strategy not available to the model) was recently discussed at the bank.

## 5. Discussion and outlook

This article develops a framework for deriving and evaluating dynamic strategies in ALM. We demonstrate that Deep ALM optimization can successfully be implemented and that the learnt strategies outperform the benchmarks. To this end, we parametrized investment and financing decisions with neural networks. The trained models comply with the regulatory constraints in almost all yield curve scenarios. The soft requirement to consistently achieve a minimum annual return is more difficult than the others and has a material impact on the performance of the bank. The choice of the time horizon *T* on which the Deep ALM model is trained influences the learnt strategies significantly. The long-term optimization with *T* = 15*y* seems to have a better alignment with the 'real' ALM problem than that with *T* = 5*y*. In most scenarios, the trained models pursue strategies with low interest rate risk. The Deep ALM model often pursues positive maturity transformations at the shorter end of the yield curve and establishes small long-term financing positions as a cheap hedge against parallel moves in the yield curve. With access to swaps, the Deep ALM model complies even better with constraints and generates higher returns-on-equity. Due to favorable market conditions for swaps, positive maturity transformation is substantiated by building up a portfolio of receiver swaps.

Deep ALM can address extremely complex and decisive questions in due course while accounting for many factors. For instance, how should a bank (re)structure its balance sheet in a negative or positive interest rate regime or when interest rates are on the verge of changing the sign, given the initial state of the balance sheet structure as well as that of the market and the current economic outlook? Deep ALM does not learn to act under all configurations but rather for a given initial state and a bundle of scenarios. This restriction is key to tame the curse of dimensionality. Furthermore, Deep ALM does not attempt to predict the future; one rather adapts best to the future while accounting for the uncertainty. Obviously, the key challenge boils down to the construction of adequate scenarios. Deep ALM is not intended to make *Asset-Liability-Committees* (ALCO) redundant. It rather creates an additional and valuable foundation for decision-making. An in-depth validation process of the learnt strategies is essential for practical purposes to comprehend the rationale behind the proposed decisions, to prevent unrealistic and extreme strategies, to get practical insights, to identify model weaknesses, and to justify strategic decisions for governance and regulatory purposes. Due to the abundance of model parameters, scenarios, and exportable quantities, sufficient resources should be allocated for these important post-training analyses. The dynamic decision-making complicates model explainability. As a matter of fact, only a small part of the model results actually produced was integrated into this publication.

The presented ALM framework has been developed iteratively with close industry collaboration. Still, our preliminary results highlight that there are remaining issues that ought to be addressed before deploying Deep ALM systems. These include the following:

– *Cutting Off the Modeling at Time* *T:* While ALM is a problem of going concern, the DSC algorithm requires the stochastic control problem to be in finite discrete time. Our results demonstrated that 'cutting off' the modeling at an arbitrary time horizon *T* is an impactful modeling decision and optimizes for strategies that are not optimal in the long run. Increasing the time horizon *T* and restricting the forward pass to a shorter window seems to be a viable way to deal with this issue. Still, there might be better approaches of solving this problem, e.g., by changing the underlying algorithm.
– *Loss Function Engineering:* Finding a loss function that reflects true preferences on the ALM strategy and its implications is challenging. Even after fixing a specific loss function, choosing relative weights of the concurrent objectives has a profound impact on the optimization problem and the learnt strategies. The weight placed on the minimum annual return constraint has a significant effect as it trades off low equity volatility versus long-term equity maximization.
– *Swaps:* The learnt strategies in the extended setting are from the viewpoint of swaps almost scenario-independent. Understanding whether the strategy of always maxing out the volume restrictions on swaps truly is a dominant learning issue is important for a practical application. Beyond that, the setup regarding swaps might be too simplistic as there is no legacy portfolio and there is a flat limit for the accessible swap volume. It is inevitable to overcome these simplifications before extending the model to longer horizons with swaps. As in the case without swaps, optimizations over longer horizons might lead to very different results compared to the 5*y* setting.

As with any model, the presented problem formulation simplifies the reality of ALM. However, a striking feature of Deep ALM is its flexibility with regard to extensions. When extending the ALM framework, one certainly has to consider the danger of overcomplicating the problem formulation. As illustrated, interpreting the learnt Deep ALM strategies is not always straightforward. In the near future, Deep ALM may not be used end-to-end, but rather as a recommender system. Explainability remains essential, which might get lost if the problem setup is unnecessarily complex. While our research may serve as a useful starting point, there are many refinements and extensions to consider when bringing Deep ALM into practice. To our mind, the most relevant extensions to the model are the following:

– *Stochastic Customer Behavior and Spreads:* The simplifying assumption that loans and deposits evolve deterministically is not necessary. The Deep ALM framework is flexible to incorporate models with *stochastic customer behavior*. Such models can also involve dependencies on the evolution of the yield curve, similar to the current depreciation mechanism, and they should take *stress scenarios* into consideration.
– *Higher Granularity:* All balance sheet positions are considered on a highly aggregated level. Especially if modeled stochastically, splitting the loan and deposit portfolios into more granular portfolios and providing these as features to the model might improve the framework.
– *Accounting Matters:* All balance sheet items and constraints are valued economically. In reality, constraints are calculated using accounting standards that deviate from *marking-to-market*. Considering the large impact of the existing frictions in this model, it seems interesting to understand what would change, if one modeled and tracked the balance sheet according to accounting standards while still using an economic valuation for assigning rewards. The necessary adjustments would require significant additional work and would make the problem computationally more expensive.
– *Yield Curve Simulation:* The experiments corroborate that the simulated yield curves carry an inductive bias in the Deep ALM framework that affects the learnt strategies. It would therefore be interesting to substitute the HJM-PCA approach with other term structure models and to quantify the model risk of the yield curve simulator; e.g., see Lütkebohmert et al. (2022). Assessing the impact of an exogenously specified *non-vanishing market price of risk* is equally important. In a next step, it would be particularly interesting to utilize “*model-independent scenarios*” based on the *signature*; e.g., see Buehler et al. (2020). Furthermore, it would be definitely worth looking at *multiple yield curves* and *defaultable bonds*; e.g., see Cuchiero et al. (2016).
– *Improvement of the Learning Process:* Getting the Deep ALM optimization to work well has been an empirical process of *trial and error*. Techniques that improved the learning process were presented in Section 3.2.3. Other approaches did not improve the performance in our implementation but might still be interesting to pursue in future work. These include, for instance, the following:

– Adjustments to the architecture of the encoding layers and the main neural network: (i) adding memory cells between the neural networks $g^{\theta_{t}}$ and $g^{\theta_{t+\Delta t}}$ and (ii) using different types of layers including convolutional layers, attention-based layers, and noisy layers (Fortunato et al., 2019) to improve *exploration*.
– Pre-training selected parts of the neural network or using *genetic optimization* to solve the credit assignment problem through the recurrent computational graph. We implemented an approach in the spirit of Ha and Schmidhube (2018), which involved pre-training prediction (LSTM) and encoder models (VAE) before learning the control *via* genetic optimizers.
– Augmenting the problem with *state* or *reward predictions*; e.g., see Silver et al. (2017).

## Data availability statement

The datasets presented in this article are not readily available because the data belongs to the bank. Requests to access the datasets should be directed to thomas.krabichler@ost.ch.

## Author contributions

All authors listed have made a substantial, direct, and intellectual contribution to the work and approved it for publication.

## Figures and Tables

**Table:** 

**Variable**	**Codomain**	**Value**	**Description**
*T*	ℕ	5 (or 15)	length of the model period [0, *T*] in years
*H*	ℕ	60 (or 180)	number of steps during the model period
Δ*t*: = *T*/*H*	ℝ_≥0_	1/12	step size of 1*m*
*T*	-	{0, Δ*t*, …, *HΔt*}	discretized model period
*t*	*T*	-	time index
*N*	ℕ	180	maximum number of steps into the future for which cash flows are modeled (at each point in the model period)
*n*	ℕ	-	number of scenarios
T	-	{Δ*t*, …, *NΔt*}	set of possible maturities of cash flows
τ	T	-	maturity index
*b* ^*B*^	ℕ	13	number of different bonds to invest in each period
*b* ^*K*^	ℕ	16	number of different bonds to finance from in each period
*s*	ℕ	6	number of different payer and receiver swaps to enter in each period
*d*	ℕ	145	feature dimension
*d*′	ℕ	64	dimension of final encoding layer

**Table:** 

**Variable**	**Codomain**	**Description**
*Y*_*t*_	ℝ^*N*^	yield curve
*D*_*t*_	ℝ^*N*^	discount factors
***Assets***		
*A*_*t*_	ℝ_≥0_	economic value of assets
*C*_*t*_	ℝ_≥0_	cash
${R^{P}}_{t}$	ℝ^*N*^	nominal cash flows of mortgages
${R^{E}}_{t}$	ℝ^*N*^	nominal cash flows of loans to individuals
$R_{t}:={R^{P}}_{t}+{R^{E}}_{t}$	ℝ^*N*^	nominal cash flows of loans
${{\tilde{R}}^{P}}_{t}$	ℝ^*N*^	nominal cash flows of new mortgages
${{\tilde{R}}^{E}}_{t}$	ℝ^*N*^	nominal cash flows of new loans to individuals
${\tilde{R}}_{t}:={{\tilde{R}}^{P}}_{t}+{{\tilde{R}}^{E}}_{t}$	ℝ^*N*^	nominal cash flows of new loans
*B*_*t*_	ℝ^*N*^	nominal cash flows from bond investments
$B_{t}^{\text{pre}}$	ℝ^*N*^	nominal cash flows from bond investments before cash flows from period *t* investments are added
***Liabilities & Equity***		
*L*_*t*_	ℝ_≥0_	economic value of liabilities
${S^{D}}_{t}$	ℝ^*N*^	nominal cash flows of non-maturing deposits
${S^{F}}_{t}$	ℝ^*N*^	nominal cash flows of term deposits
$S_{t}:={S^{D}}_{t}+{S^{F}}_{t}$	ℝ^*N*^	nominal cash flows of deposits
${{\tilde{S}}^{D}}_{t}$	ℝ^*N*^	nominal cash flows of new non-maturing deposits
${{\tilde{S}}^{F}}_{t}$	ℝ^*N*^	nominal cash flows of new term deposits
${\tilde{S}}_{t}:={{\tilde{S}}^{D}}_{t}+{{\tilde{S}}^{F}}_{t}$	ℝ^*N*^	nominal cash flows of new deposits
*K*_*t*_	ℝ^*N*^	nominal cash flows from bond financing
$K_{t}^{\text{pre}}$	ℝ^*N*^	nominal cash flows from bond financing before cash flows from period *t* financing are added
*E*_*t*_	ℝ_≥0_	economic value of equity
***Decision Variables***		
$a_{t}^{B}$	${\mathbb{R}}_{\geq 0}^{b^{B}}$	number of bonds to buy at time t for each available maturity
$a_{t}^{K}$	${\mathbb{R}}_{\geq 0}^{b^{K}}$	number of bonds to issue at time t for each available maturity
$a_{t}^{pay}$	${\mathbb{R}}_{\geq 0}^{s}$	number of payer swaps to enter into at time t for each available maturity
$a_{t}^{rec}$	${\mathbb{R}}_{\geq 0}^{s}$	number of receiver swaps to enter into at time t for each available maturity
*a*_*t*_	${\mathbb{R}}_{\geq 0}^{b^{B}+b^{K}}$	concatenated decision vector
*X*_*t*_	ℝ^*d*^	features
***Other Cash Flow Variables***		
*r*_*t*_	ℝ	received interest payments on all loans
$Z_{t}^{\alpha_{i}^{B}}$	ℝ^ℕ^	cash flows received on the *i*^*th*^ investment bond issued at time *t* for *i* ∈ {1, 2, …, *b*^*B*^}
$Z_{t}^{\alpha_{i}^{K}}$	ℝ^ℕ^	cash flows received on the *i*^*th*^ financing bond issued at time *t* for *i* ∈ {1, 2, …, *b*^*K*^}
*u* ^*D*^	ℝ	interest rate paid on non-maturing deposits
*u* ^*F*^	ℝ	interest rate paid on term deposits
*I* ^*D*^	ℝ^*N*^	interest payments made on non-maturing deposits for each associated maturity
*I* ^*F*^	ℝ^*N*^	interest payments made on term deposits for each associated maturity
*c*_*t*_	ℝ	material and personnel cost
*cp*_*t*_	ℝ	penalty for holding cash when interest rates are negative
*CF*_*t*_	ℝ	cash flow from loans, deposits, and costs
δ_*t*_	ℝ	dividend
$h_{t}^{pay}$	ℝ^*s*(*H*−1)^	payer swap holding portfolio in units of swaps
$h_{t}^{rec}$	ℝ^*s*(*H*−1)^	receiver swap holding portfolio in units of swaps
$V_{t}^{fix}$	ℝ^*s*(*H*−1)^	value of outstanding fixed payments of all swaps at time *t*
$V_{t}^{float}$	ℝ^*s*(*H*−1)^	value of outstanding floating payments of all swaps at time *t*
$V_{t}^{spread}$	ℝ^*s*(*H*−1)^	value of outstanding spread payments of all swaps at time *t*
*N* ^*A*^	ℝ_≥0_	net swap assets (value of swap portfolio if positive)
*N* ^*L*^	ℝ_≥0_	net swap liabilities (absolute value of swap portfolio if negative)
$\eta_{t}^{pay}$	ℝ	cash flow from all held payer swaps at time *t*
$\eta_{t}^{rec}$	ℝ	cash flow from all held receiver swaps at time *t*
***Spreads & Growth Factors***		
κ_*L*_	ℝ	annual spread on loans
κ_*B*_	ℝ	annual spread on investments
κ_*K*_	ℝ	annual spread on borrowings
κ_*S*_	ℝ	annual spread on swaps
ρ_*L*_	ℝ	annual growth of loans
$\rho_{S^{D}}$	ℝ	annual growth of non-maturing deposits
$\rho_{S^{F}}$	ℝ	annual growth of term deposits
***Loss Function***		
γ	ℝ_≥0_	risk aversion coefficient
μ	ℝ_≥0_	annual return target
$P_{t}^{i}$	ℝ_≥0_	penalty of a violation of the *i*^*th*^ constraint at time *t* for *i* = 1, 2, …, 6
σ_*i*_	ℝ_≥0_	weight of violation penalty on *i*^*th*^ constraint for *i* = 1, 2, …, 6
*p*	ℝ_≥0_	penalty accumulated across all constraint violations in the model period
λ	ℝ_≥0_	weight of penalty loss in total loss

## Footnotes

^1^Data Source: Swiss National Bank, https://data.snb.ch/en/topics/ziredev/cube/rendopar.

^2^In the entire article, all balance sheet items are valuated in *economic* terms. For other accounting standards, the framework would have to be adjusted accordingly.

^3^Duration denotes the cash flow weighted time in years to the settlement dates. Mathematically, it coincides with the valuation sensitivity toward interest rate changes; see Mazzi (2013, Appendix D). The capital commitment period is not necessarily identical to the fixed-interest period.

^4^This is done because HJM yield curve dynamics are generally not required to be Markovian. In our experiments, we simulate Markovian yield curve dynamics, which makes this technicality irrelevant.

^5^Variants of this type of algorithm appear in many applied machine learning fields under different names. In the field of deep hedging, it is sometimes referred to as *periodic policy search* (Buehler et al., 2022b).

^6^Simple constraints (e.g., on the output range of the controls *a*_*t*_) can often be accounted for by adding a scaling operation (e.g., softmax) to the final output.

^7^In reality, inflows are indirectly influenced *via* the economic circle as well as the bank's interest rate policy. Another important model extension includes the consideration of stress scenarios concerning deposits.

^8^Some banks impose maximal allowances and waiting periods for the withdrawal of large volumes.

^9^In our experiments, we choose *b*^*B*^ = 13 corresponding to investment maturities of 3*y*–15*y* and *b*^*K*^ = 16 corresponding to financing maturities of 3*m* and 1*y*–15*y*.

^10^1 basispoint (bps) refers to as 0.01%. The considered retail bank is a *price taker*, in contrast to a *market maker*. The spread constitutes the service fee that the bank has to bear for any transaction.

^11^In the sense of von Neumann and Morgenstern (1947).

^12^This does not mean that the minimum return constraint has the smallest impact on the penalty component of the loss. On the contrary, we will later find that this constraint is more difficult to comply with than others; see Section 4.1.

^13^We use the convention that $a_{0}^{pay}$ is added to the first *s* entries of $h_{t}^{pay}$, $a_{1}^{pay}$ is added to entries *s*+1 until 2*s*, and so forth.

^14^A short payer swap is a receiver swap, and *vice versa*. This restriction is imposed to prevent redundancies in the action space.

^15^The issue that one cannot take the forward difference for the last entry of *F*_*t*_ is solved by starting with a higher-dimensional forward curve, where the additional entries correspond to larger maturities, and reducing the dimensionality of *F*_*t*_ step-by-step.

^16^This choice is for computational reasons. In the previous period, the constrained quantities have already been computed; see Algorithm 1. These values should be reasonably close to the actual values after the balance sheet has been rolled over.

^17^Alternatively, learning investments and borrowings in excess of reinvested investment of financing positions also works well.

^18^Following the definition in Oord et al. (2018), the operator *sg* is defined as identity at forward computation time and has zero partial derivatives, thus effectively constraining its operand to be a non-updated constant.

^19^The code is available at https://github.com/konmue/deep_alm; for data privacy reasons, the repository is private at the time of the publication of this article. We intend to release the core scripts in due course.

^20^Losses only occur if the negative slope offsets the spreads that the bank charges on both sides of its business model: charging more interest on loans and paying less interest on deposits.

^21^The analysis on the 15*y* horizon is omitted because the volume requirements on swaps have been set with the 5*y* horizon in mind. The approximation of unhedged assets and liabilities, which restricts the volume of the swap portfolios, is likely too simplistic when optimizing for a longer time horizon.

## Notation

The following appendix provides an overview of the notation for the formulation of the ALM optimization problem introduced in Section 2. Variables that are used exclusively in the introduction are not listed here.
